# Connexin-46/50 in a dynamic lipid environment resolved by CryoEM at 1.9 Å

**DOI:** 10.1038/s41467-020-18120-5

**Published:** 2020-08-28

**Authors:** Jonathan A. Flores, Bassam G. Haddad, Kimberly A. Dolan, Janette B. Myers, Craig C. Yoshioka, Jeremy Copperman, Daniel M. Zuckerman, Steve L. Reichow

**Affiliations:** 1 Department of Chemical Physiology and Biochemistry, Oregon Health and Science University, Portland, OR 97239 USA; 2 Department of Chemistry, Portland State University, Portland, OR 97201 USA; 3 Department of Biomedical Engineering, Oregon Health and Science University, Portland, OR 97239 USA; 4 Present Address: Biophysics Graduate Group, University of California, Berkeley, CA 94720 USA

**Keywords:** Ion transport, Membrane structure and assembly, Permeation and transport, Cryoelectron microscopy, Molecular modelling

## Abstract

Gap junctions establish direct pathways for cells to transfer metabolic and electrical messages. The local lipid environment is known to affect the structure, stability and intercellular channel activity of gap junctions; however, the molecular basis for these effects remains unknown. Here, we incorporate native connexin-46/50 (Cx46/50) intercellular channels into a dual lipid nanodisc system, mimicking a native cell-to-cell junction. Structural characterization by CryoEM reveals a lipid-induced stabilization to the channel, resulting in a 3D reconstruction at 1.9 Å resolution. Together with all-atom molecular dynamics simulations, it is shown that Cx46/50 in turn imparts long-range stabilization to the dynamic local lipid environment that is specific to the extracellular lipid leaflet. In addition, ~400 water molecules are resolved in the CryoEM map, localized throughout the intercellular permeation pathway and contributing to the channel architecture. These results illustrate how the aqueous-lipid environment is integrated with the architectural stability, structure and function of gap junction communication channels.

## Abstract (web summary)

The local lipid environment is known to affect the structure, stability and intercellular channel activity of gap junctions, however, the molecular basis for these effects remains unknown. Here authors report the CryoEM structure of Cx46/50 lipid-embedded channels, by which they reveal a lipid-induced stabilization to the channel.

## Introduction

The connexins are a family of transmembrane proteins (21 isoforms in human) that form intercellular channels for cell-to-cell communication^1,2^. These intercellular channels establish a ~1.4 nm pore that couples the cytoplasms of neighboring cells and enable direct passage of electrical and small-molecule signals (such as ions, second messengers, hormones, and metabolites)^3^ and therapeutic agents^4^. Tens to 1000s of connexin channels may assemble together to form large hexagonally packed arrays, a.k.a., plaques, known as gap junctions. In this way, gap junctions enable the near instantaneous response of electrical synapses in the brain and heart, and contribute to the long-range signaling and metabolic coupling of most tissues. Because of these fundamental roles, aberrant gap junctional coupling is associated with a variety of human diseases, including blindness, deafness, skin disorders, arrhythmia, stroke, and cancers^5–7^.

Gap junction intercellular communication is facilitated by a unique macromolecular architecture, where intercellular channels directly couple the cytoplasms of two neighboring cells. The lipid bilayers of opposing cells are separated by a characteristic gap of ~3.5 nm^8^, a feature for which these structures were first recognized in electron micrographs of cell sections^9,10^. Furthermore, large-scale gap junctional plaque formation is dependent upon a dense mosaic of protein–lipid interactions. In vitro reconstitution studies have established that plaque assembly and intercellular channel function are dependent on the lipid environment^10–15^. However, the molecular basis for these effects remain largely unknown, due to the lack of high-resolution structural information within a lipid bilayer.

Here we present an electron cryo-microscopy (CryoEM) structure of native connexin-46/50 (Cx46/50) intercellular channels stabilized in a dual lipid nanodisc system at 1.9 Å resolution—providing a near-atomic level of detail for this class of membrane channels. These structural results are coupled with all-atom molecular dynamics (MD) simulation studies, which together reveal many architectural and proposed functional features of the connexin channels. Cx46/50 is shown to have a remarkable influence on the local lipid environment, effectively inducing a phase separation (to the gel state) that is specific to the extracellular lipid leaflet of the two opposed membranes. Three-dimensional (3D) heterogeneity analysis of the CryoEM data identified multiple lipid configurations that co-exist within the dynamic lattice of stabilized lipids, which is further detailed by MD. In addition, ~400 water molecules are resolved in the CryoEM map, localized at architectural and functionally important sites. Together, this work uncovers previously unrecognized roles of the aqueous-lipid environment in stabilizing the structure and assembly of the gap junctions, and suggest Cx46/50 plays an important role in shaping the properties of local membrane environment.

## Results

### Structural overview of Cx46/50 in a lipid environment

Native (heteromeric/heterotypic) Cx46/50 intercellular channels were purified from mammalian lens tissue (obtained from sheep)^16^. Freshly purified channels were reconstituted into self-assembling lipid nanodiscs containing pure dimyristoyl phosphatidylcholine (DMPC) at room temperature (~25 °C), supported by the membrane scaffold protein 1E1 (MSP1E1)^17^ (see “Methods”). Under optimized conditions, the reconstitution resulted in a monodispersed population of intercellular channels embedded into a pair of lipid nanodiscs, as assessed by size-exclusion chromatography (SEC) and negative-stain electron microscopy (EM) (Supplementary Fig. 1).

**Fig. 1:**
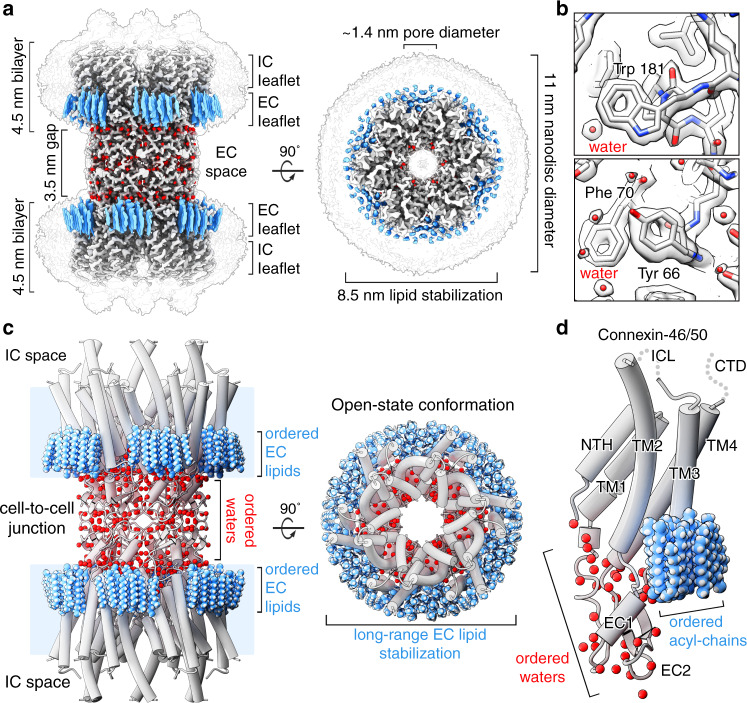
Structure of connexin-46/50 in lipid nanodiscs by CryoEM. **a** CryoEM 3D reconstruction of Cx46/50 (white) in an open-state conformation, with resolved lipid acyl chains (blue) and water molecules (red). Transparent silhouette displays the map at low contour to illustrate the dimensions of the lipid nanodisc densities, with intracellular (IC) and extracellular (EC) lipid leaflets indicated. **b** Zoom views of the CryoEM map and fitted atomic models, showing high-resolution features observed at 1.9 Å resolution. **c** Model of Cx46/50 (cylinder representation) with extracellular (EC) lipids and ordered water molecules displayed (spheres). **d** Cx46/50 monomer, 15 bound lipids, and 33 waters associated with each subunit. Domains labeled for transmembrane helices (TM1–4), extracellular loops (EC1–2), and N-terminal helix (NTH). The intracellular loop (ICL) and C-terminal domain (CTD) are not resolved, indicated by dotted lines.

Structure determination by high-resolution single-particle CryoEM resulted in a high-quality 3D reconstruction, with an overall resolution of 1.90 Å (gold-standard Fourier-shell correlation (FSC)) (Fig. 1a, b, Supplementary Figs. 2 and 3, and Supplementary Movie 1). The quality of the CryoEM map allowed for a detailed stereochemical structural refinement of both Cx46 and Cx50 (Fig. 1b, Supplementary Table 1, and Supplementary Fig. 3). The heteromeric pattern(s) of Cx46/50 co-assembly remain unresolved, following various attempts at computational image classification (see “Methods”). Nevertheless, atomic models of both Cx50 and Cx46 isoforms were equally well-fit into the D6-symmetrized CryoEM map, reflecting their close sequence and structural similarities, 89% sequence similarity over the structured regions, and a resulting 0.17 Å backbone root-mean-squared deviation (r.m.s.d.) (see “Methods” and Supplementary Fig. 3 for details and limitations regarding the heterogeneity of the natively isolated specimen).

Cx46/50 is captured in the stabilized open state, as previously described^16^ (backbone Cα r.m.s.d. = 0.49–0.56 Å and Supplementary Fig. 5), and exposes many undescribed features of the connexin channels that are detailed below. Intercellular channels are constructed by a dodecameric (12-mer) assembly, with six subunits assembled into “hemi-channels” that dock together through extracellular domains, resulting in a continuous ~1.4 nm pore for intercellular permeation (Fig. 1a, c and Supplementary Movie 2). The distance separating the two lipid nanodisc densities is ~3.5 nm (Fig. 1a, c), matching that observed by X-ray diffraction on native gap junctional plaques^8^.

Each monomer consists of four transmembrane helices (TM1–4), two extracellular loops (EC1–2) that form the sites of docking interaction, and an amphipathic N-terminal helix (NTH), implicated in channel selectivity/gating. The NTH is well resolved in the stabilized open state, as we have previously described^16^ (Fig. 1c, d and Supplementary Movie 3). However, the significant enhancement in resolution allowed for a detailed refinement of side chain conformations and notable improvement in precision at functional sites, including the NTH domain and the EC1/2 docking sites (Supplementary Figs. 3 and 5). Furthermore, the quality of the CryoEM map allowed for modeling previously unresolved regions of TM2 and TM3, which effectively extend the cytoplasmic vestibule of the channel by ~20 Å, as compared to our previous model (Fig. 1c, d), significantly augmenting the electrostatic environment of the pore entrance (Supplementary Fig. 5). The intracellular loop (ICL) and C-terminal domain (CTD) remain unresolved, presumably due to intrinsic disorder of these regulatory domains^16,18,19^.

Perhaps the most remarkable features of the CryoEM map, however, are the non-protein components of the cell-to-cell junction that are now resolved. A bouquet of 15 ordered lipid acyl chains is held in place by each of the 12 connexin subunits, which appear to buttress the channel assembly by filling a cavity formed at the lateral subunit interfaces (Fig. 1a, c, d; blue). Surprisingly, acyl-chain densities are observed well beyond the first layer of annular lipids that directly interact with the TM domains (primarily TM4 and TM3 of a neighboring subunit) (Fig. 1c, d and Supplementary Fig. 6), suggesting Cx46/50 has a long-range effect on the stability and biophysical properties of the membrane. Remarkably, all of the resolved lipid densities in the CryoEM map are specifically localized to the extracellular leaflet of the bilayer, indicating a selective interaction with the local lipid environment. In contrast, individual lipids are not resolved in the intracellular leaflet, even at lower contour levels (Fig. 1a and Supplementary Fig. 2) presumably due to intrinsic disorder and/or lack of specific interaction with this region of the channel.

**Fig. 2:**
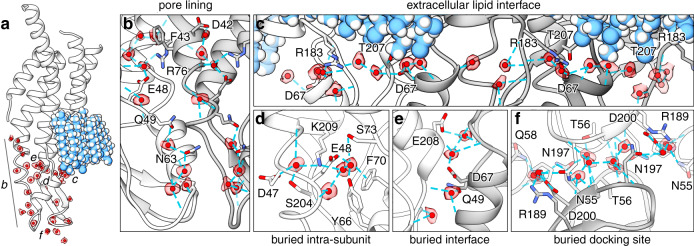
Ordered water molecules resolved in Cx46/50 by CryoEM. **a** Cx46/50 subunit with segmented CryoEM density of waters overlaid in transparency (colored as in Fig. 1). Labels in **a** indicate position of the various zoom views, presented in **b–f**, showing water molecules bound to **b** pore-lining sites, **c** extracellular lipid interface, **d** buried intra-subunit sites, **e** buried subunit interface sites, and **f** buried cell-to-cell docking sites. In **b**–**f**, amino-acid sidechains forming hydrogen bonds to water are displayed (blue dashed lines) and labeled using Cx50 numbering.

In addition to stabilized lipids, 396 ordered water molecules are resolved throughout the channel (33 waters per subunit) (Figs. 1 and 2 red, and Supplementary Fig. 6). Waters are found at both solvent accessible and buried sites within the core of the channel, apparently contributing to the permeation pathway and structural integrity of the channel assembly (Figs. 1 and 2). The assignment of water densities was validated by assessment of hydrogen-bonding patterns (<4 Å donor–acceptor distances) and supported by comparison to all-atom equilibrium MD simulations conducted in the presence of explicit water and 150 mM NaCl or KCl (see “Methods” and Supplementary Figs. 7 and 8). There was no clear evidence that the resolved solvent sites correlated with low-affinity-ion-binding sites observed by MD^16^. In the following sections, we describe these resolved features in further detail and discuss their potential structural and functional roles.

### Roles of stabilized waters in Cx46/50 intercellular channels

Gap junctions establish aqueous pathways that allow a variety of cytosolic substrates, less than ~1 kDa in size, to permeate from cell to cell^20^. The permeation pathway is established by the pore-lining NTH domain, TM1/2, and EC1 domains (Fig. 1c, d). Within the channel pore of Cx46/50, there are 108 waters bound at solvent-exposed sites (9 per subunit). Pore-bound waters localize to regions of the EC1 domain and TM1 parahelix, and mediate an extensive network of h-bonding interactions, involving D42, F43 (π-bonding), E48, Q49, N63, and R76 in Cx50 (positions 42 and 43 are Glu in Cx46), and several protein backbone interactions (Fig. 2a, b and Supplementary Fig. 6).

EC1 and the TM1 parahelix contribute to the selectivity, conductance, and slow (loop) voltage-gating mechanisms of Cx46/50^16,21–23^ and other connexins^24–29^, and are implicated in Ca^2+^ regulation in Cx26 by X-ray crystallography^19^, MD studies^27,30^, and by functional mutation studies of Cx46^30^. As such, these pore-lining waters may functionally contribute to these mechanisms, e.g., by orienting or extending the hydrogen-bonding potential of amino-acid sidechains involved in the coordination of substrates (or regulatory ions), buffering the electrostatic properties of the channel pore, or integrating the electrostatic network that is proposed to couple EC1/TM1 to the fast (NTH) voltage-gating domain^31,32^.

On the extracellular surface of the channel, symmetry-related rings of tightly bound water molecules are organized at the extracellular aqueous-lipid boundary (Figs. 1a, c and 2a, c). In the ensemble CryoEM map, the phosphatidylcholine (PC) lipid head groups are not resolved (due to local disorder described in the following sections). Nevertheless, these stabilized rings of water are nominally positioned at the acyl-head group boundary of the extracellular lipid leaflet. These waters are stabilized by hydrogen bonds with EC1/2 residues (D67, R183/Q171, and T207/T195 in Cx50/Cx46) and expected to be further coordinated through nonspecific interactions with the phospho-glycerol backbone of the extracellular PC lipids (Fig. 2c; and discussed below).

The EC1/2 domains appear to be the most well-ordered region of the channel, as reflected by local resolution of the CryoEM density map (Supplementary Fig. 3) and root-mean-square fluctuation (r.m.s.f.) analysis of MD trajectories (Supplementary Fig. 7) This high-degree of stability reflects the important functional role of the EC1/2 domains in maintaining an electro-chemical seal at the cell-to-cell junction. Several clusters of water molecules are found buried a sites located both within and between the EC1/2 domains of individual subunits (Fig. 2a, d-f). A cluster of four waters are buried within the EC1/2 domains coordinated by residues D47, E48, Y66, F70 (π bonding), S73, S204/S192, and K209/K197 in Cx50/46 (Fig. 2d). Four additional waters are buried at the lateral EC domain interface formed by neighboring subunits, primarily coordinated by hydrogen-bonding interactions with the peptide backbone and sidechains of Q49, D67, and E208/E196, in Cx50/Cx46 (Fig. 2e). The degree of coordination of these buried waters suggest they contribute to the architectural integrity of EC1/2 docking domains and may in part explain why deleterious mutations at D47, E48, and D67 in Cx50, linked to cataract formation, disrupt junctional coupling and/or biogenesis^33–35^.

The EC1/EC2 domains also play important roles in establishing the specificity of hemi-channel docking interactions formed between different connexin isoforms and the ability to establish the so-called homotypic or heterotypic channels^36–38^. Elucidating the determinants of hemi-channel recognition is therefore critical to understanding the principles dictating cell-type specificity of gap junctional coupling^39^. It has been proposed that isoform-specific hydrogen-bonding patterns that bridge the EC1/EC2 interface govern hemi-channel docking compatibility^38,40^. Contributing to this bridging site in Cx46/50 is a cluster of 12 water molecules (per subunit pair) that are deeply integrated within a dense network of hydrogen bonds between EC1/EC2 residues of opposed subunits (Fig. 2f). At the center of this network is the highly conserved K/R-N-D motif found in EC2 of Group I compatible connexins (including Cx50, Cx46, Cx32, and Cx26). Genetic mutations of this motif in Cx46/50 are linked to congenital cataracts^16,41^, as well as other genetic disorders (e.g., Charcot-Marie-Tooth disease^42^ and non-syndromic deafness^43^), when mutated in other Group I connexins. These observations suggest interfacial waters may play previously unappreciated and functionally important roles in establishing the structural integrity of the intercellular channel and contribute to the specificity of hemi-channel docking interactions involved in regulating the formation of intercellular communication pathways.

### Long-range ordering of the extracellular lipid leaflet

**Fig. 3:**
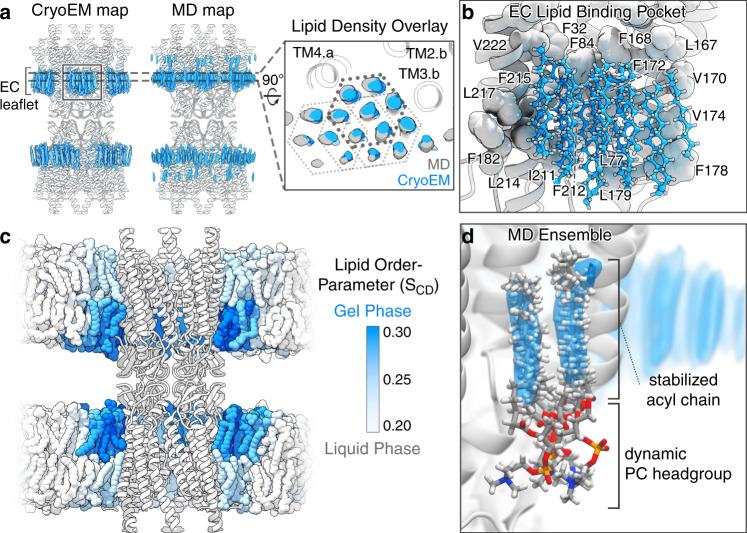
Cx46/50 induces a local phase separation to the extracellular lipid leaflet. **a** Comparison of acyl-lipid density maps (blue) obtained by CryoEM and time-averaged all-atom MD simulation, overlaid onto the Cx46/50 ribbon structure (white). Inset shows a slice view (rotated 90°) of overlaid acyl-lipid densities by CryoEM (blue) and MD simulation (gray). The hexagonal packing pattern of acyl chains is indicated (solid and dotted lines) and TM helices interacting with lipid are labeled. Different subunits are indicated by suffix (*a* or *b*). **b** Zoom view of the acyl-lipid-binding pocket, with lipid-binding residues displayed (spheres) and labeled (Cx50 numbering). **c** MD snapshot of Cx50 in phosphatidylcholine (PC) lipid bilayers, with time-averaged lipid order parameter (*S*_CD_) for each lipid indicated by shading (blue = 0.30 to white = 0.20). **d** Zoom view, showing an ensemble super-positioning of symmetry-related lipids obtained by MD simulation (displayed as all-atom representation, C = gray, H = white, O = red, N = blue, P = orange) occupying the MD-based lipid acyl-chain density map (blue).

The degree of long-range stabilization to the local lipid environment observed in the Cx46/50 nanodisc reconstruction is extraordinary, extending several solvent layers away from the protein. DMPC was selected as a model lipid because of the high PC content of mammalian (sheep) lens^44^, and reconstitution studies show DMPC produces Cx46/50 assemblies that are indistinguishable from those formed with native lipids^14,45^. Due to its complete saturation DMPC has a relatively high phase transition (i.e., melting) temperature (*T*_m_) compared to other biological lipids (*T*_m_ ~24 °C in pure lipid vesicles^46^). This value is close to the temperature at which reconstitution was performed (~25 °C, room temperature). However, in nanodiscs the melting temperature of DMPC is reportedly higher (~28 °C), due to compartmentalization effects by the MSP scaffold^47^. Nevertheless, the specific localization of stabilized lipids to the extracellular leaflets observed by CryoEM (and also by MD studies, described below) suggested long-range lipid stabilization is induced through interactions with Cx46/50 (Figs. 1 and 3a).

To gain further insight into the lipid stabilization observed by CryoEM, we analyzed time-averaged densities of DMPC acyl-chain positions obtained by unbiased all-atom MD simulations for both Cx50 and Cx46, conducted at 37 °C, where the starting positions of DMPC molecules had been randomly placed into a 15.4 × 15.4 nm lipid bilayer (see “Methods” and Supplementary Fig. 7a). Following equilibration, the resulting acyl-lipid density profiles displayed remarkable similarity to what was resolved by CryoEM (Fig. 3a). In both cases, lipids within the extracellular leaflets are specifically stabilized, as compared to the intracellular lipid leaflet (Fig. 3a). Furthermore, the resolved clusters of acyl-chain densities obtained by MD display the same hexagonal packing pattern that extends three to four orders beyond the annular shell, as observed by CryoEM (Fig. 3a, inset).

The corroborating results obtained by MD imply that the lipid stabilization observed by CryoEM is directly induced by structural features of the Cx46/50 TM domains and not an artifact of the nanodisc. Each cluster of lipids is bound by a shallow pocket of hydrophobic and aromatic residues, displayed by TM2/3 and TM4 of adjacent subunits (Fig. 3a, b). A cleft, rich in aromatic sidechains (formed by F32, F84, L167/L155, and F168/F156 in Cx50/C46), intercalates into the bilayer, appearing to bisect the extracellular leaflet from the more disordered intracellular leaflet (Fig. 3b). In this way, it appears that the acyl-lipid-binding pocket selectively grasps a large bouquet of lipids from the extracellular leaflet, inducing long-range stabilization to the membrane through extensive Van der Waals interaction.

The extended acyl-lipid chain conformation and hexagonal packing adopted by the bouquet of bound lipids are indicative of a quasi phase transition to the liquid-ordered (or gel-like) state. To obtain a more quantitative assessment of the degree of lipid stabilization, we extracted SN1 and SN2 lipid order parameters (*S*_CD_) from the MD simulations, which have been parameterized to fit well to experimental NMR-based order parameters^48^. These results are consistent with the notion that lipids in the intracellular leaflet are maintained in a fluid state, whereas Cx46/50 induces a phase transition from a fluid to a gel-like state that is specific to the extracellular lipid leaflet, as indicated by a shift in order parameters to above ~0.25^49^ (acyl-chain carbons 4–11; Fig. 3c and Supplementary Fig. 9), which extend ~10–20 Å from the protein surface, as observed by CryoEM.

Although this degree of stabilization to the local lipid environment is likely to depend on lipid type, the general effects may be functionally important. For example, by contributing to the architectural integrity at the gap junctional interface, partitioning specific types of lipids, or even templating long-range hexagonal packing interactions found in plaque assemblies^45,50^. In this context, it is noteworthy that connexins localize to lipid raft domains^51,52^, which are rich in high *T*_m_ lipids (e.g., sphingomyelin) and characterized as forming a liquid-ordered state.

### Dynamic lipid configurations captured by CryoEM and MD

**Fig. 4:**
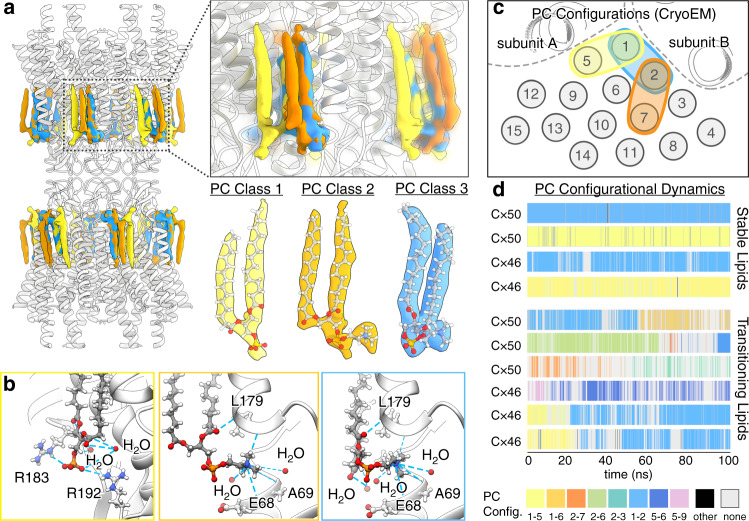
PC lipid configurational heterogeneity and dynamics resolved by CryoEM and MD. **a** Segmented phosphatidylcholine (PC) density maps obtained by CryoEM 3D heterogeneity analysis and classification (PC Class 1—yellow, PC Class 2—orange, PC Class 3—blue). Insets show a zoom view displaying the overlapping features of resolved lipid configurations and segmented densities with fitted atomic models obtained from the three PC classes. CryoEM density for all other non-unique acyl-lipid chains, with unresolved head groups, have been omitted for clarity. **b** Zoom view showing Cx50 hydrogen bond interactions (blue dashed line) between the PC lipid head group and phospho-glycerol backbone. Interacting amino acids and stabilized water molecules are labeled. Yellow box—PC Class 1, orange box—PC Class 2, blue box—PC Class 3. **c** Illustration showing acyl-chain positions and configurational assignments resolved by CryoEM (represented as gray circles and numbered 1–15). **d** PC configurational classification and dwell times obtained by all-atom MD simulation, showing representative populations of stable (non-transitioning) and dynamic (transitioning) lipids. PC configurations were classified by acyl-chain occupancy in densities numbered as in **c** and colored uniquely (as indicated, bottom of **d**).

Another notable feature of the lipid densities observed in the CryoEM map is that PC head groups are not observed, despite sufficient resolution to expect such features (Figs. 1 and 3a). Super-positioning of representative lipid conformations obtained by MD show that, although the annular lipid acyl-chains were relatively well ordered and superimpose, their corresponding head groups remain conformationally dynamic and/or heterogeneously positioned (Fig. 3d and Supplementary Movie 4). Such behavior would rationalize the lack of resolvability in the averaged CryoEM density map. In an attempt to resolve this heterogeneity, we conducted 3D classification analysis on the ensemble CryoEM data (“Methods” and Supplementary Fig. 4), which resulted in three distinct 3D reconstructions resolved at resolutions of ~2.5 Å (gold-standard FSC) (Fig. 4a-c and Supplementary Fig. 4).

In each of the 3D classes, PC head groups and/or phospho-glycerol backbone of individual annular lipids were uniquely resolved (PC Class 1–3, Fig. 4a). The conformational state of Cx46/50 is very similar in all three classes, and essentially indistinguishable from models derived from the ensemble density map (Cα r.m.s.d.’s = 0.24–0.34 Å). Notably, structural features of each of these fully resolved lipids are shared among these classes. For example, the SN2 acyl chain of PC Class 1 overlays with the SN1 chain of PC Class 3 (Fig. 4a, c; yellow and blue). Likewise, the SN1 chain of PC Class 2 also overlays with the SN1 chain of PC Class 3 (Fig. 4a, c; orange and blue). This suggested that multiple, overlapping, configurational states are capable of supporting the same lattice of acyl-chain positions observed in the CryoEM reconstructions, consistent with nonspecific and/or transient binding interactions.

The choline head group of PC Class 1 remained unresolved; however, the negatively charged phospho-glycerol backbone is clearly visualized and appears to be stabilized by flanking positively charged arginine residues R183 and R192 in Cx50 (Q171 and R180 in Cx46) (Fig. 4b; left), and hydrogen bonding with two water molecules (part of the belt of extracellular waters described above, see Fig. 2c). PC Class 2 and 3 resolve distinct acyl-chain configurations, yet, both of these states share a similar placement of their positively charged choline head groups. Head group placement of these lipids is supported by nonspecific hydrogen bond interactions with backbone carbonyls presented by EC1 and TM3 (involving residues E68, A69, and L179 in Cx50; position 68 is an Arg in Cx46) and a buried water molecule (Fig. 4b; center and right). The phospho-glycerol backbones of PC Class 2/3 lipids are coordinated by hydrogen bonding to local waters and the backbone amide of L179 in Cx50 (L167 in Cx46). Remarkably, the SN2 acyl chain and glycerol backbone of the PC Class 2 lipid is completely resolved, despite lacking any direct contact with the Cx46/50 protein interface (Fig. 4a, c).

Collectively, these observations support the notion that although the Cx46/50 acyl-chain interactions appear to be high affinity, the lipid head group interactions are nonspecific and adopt a variety of configurational/conformational states. This is reinforced by our MD simulation studies for both Cx50 and Cx46, where mapping of PC arrangements at resolved acyl-chain densities show a variety of configurational states that co-exist within the dodecameric assembly. Furthermore, during the timescale of the simulations (100 ns), time-resolved PC configurations could be classified as being either stable or dynamically transitioning between multiple configurational states (Fig. 4d and Supplementary Movie 5, 6). Notably, the most stable (yet overlapping) configurations (e.g., 1–2 and 1–5 configurations) are the same as those resolved by CryoEM 3D classification (Fig. 4a, c, d, blue and yellow, respectively). Yet, other lipid trajectories were observed interconverting between these same configurations over this relatively short timescale (Fig. 4d). The degree of configurational preference diminishes beyond the first two solvent shells, presumably due to the loss of energetic influence induced by protein interactions (Fig. 4d), and reflect the randomized head group arrangements expected of a bulk lipid population. Taken together, these data show Cx46/50 stabilizes the dynamic local lipid environment through nonspecific interactions with the extracellular leaflet, with multiple configurational PC lipid states existing at the annular interface and effectively captured by CryoEM.

## Discussion

The structure and function of membrane proteins are deeply integrated with their lipid environment. Our mechanistic understanding of protein–lipid interactions has been largely shaped by high-resolution structures of membrane proteins where specifically bound lipids have been captured at well-defined binding sites^53^. Yet, most interactions made between membrane proteins and their local membrane environment are relatively nonspecific and highly dynamic. The mechanistic principles and biophysical consequences underlying such interactions remains poorly understood, as these interactions are typically lost during protein purification, or remain too dynamic to resolve by traditional structural methods. By exploiting the potential of lipid nanodisc technologies coupled with single-particle CryoEM and MD simulation, we show that Cx46/50 intercellular communication channels form dynamic interactions with annular lipids. These nonspecific interactions have long-range stabilizing effects capable of inducing a phase separation to high *T*_m_ lipids, which may extend ~20 Å from the protein surface. These interactions appear selective toward the extracellular leaflet of pure PC membranes, which may have significant consequences on the biomechanical properties and lipid composition of gap junctional domains. In fact, the lack of resolved lipids in the intracellular leaflet may reflect the selectivity at this leaflet toward non-PC lipid types, as suggested for Cx26/32^15^. The methods developed here provide a valuable high-resolution platform for developing our deeper understanding of the specificity and physiological role lipids play in gap junction biology, and how aberrant lipid environments may contribute to connexin-related pathologies. Indeed, the capability of resolving connexin channels beyond the critical threshold of ~2.0–2.5 Å resolution, the precision desired for structure-based drug design—e.g., providing detailed stereochemical models and placement of architectural water molecules—now opens the door to rational development of selective high-affinity pharmacological tools that are desperately needed in this field to better understand and potentially treat a wide range of connexin-opathies^54^.

## Methods

### MSP expression and purification

A plasmid containing the coding sequence for MSP1E1 was obtained from Addgene^17^ and the protein was expressed and purified as described^55^, with minor modification. Freshly transformed *Escherichia coli* cells (BL21Gold-DE3, Agilent, Catalog #230130) were grown in LB medium containing 50 μg mL^−1^ kanamycin at 37 °C with shaking (250 r.p.m.). Induction with 0.5 mM isopropyl β-d-1-thiogalactopyranoside was performed when OD_600_ reached 0.5–0.6 and allowed to express for 3–5 h post induction at 37 °C. Cells were collected by centrifugation at 4000 × *g* for 20 min at 4 °C and cell pellets were resuspended in MSP Lysis Buffer (40 mM Tris pH 7.4, 1% Triton X-100, 1 mM phenylmethylsulfonyl fluoride (PMSF)) at a density of ~20 mL of Lysis Buffer per liter of culture. Cell suspensions were flash frozen in liquid nitrogen and stored at −86 °C for up to several months.

Frozen cell suspensions were thawed from −86 °C storage, supplemented with 1 mM PMSF, and lysed by sonication on ice. Crude lysate was cleared by ultra-centrifugation at 146,550 × *g* for 30 min at 4 °C. The supernatant was filtered (Millipore; 0.22 μm) and applied to a gravity column with 5 mL of HisPur Ni-NTA resin (Thermo Fisher Scientific) prepared in equilibration buffer (40 mM Tris pH 7.4). MSP-bound resin was washed with five column volumes (CV) of equilibration buffer, followed by five CVs of each of the following: Triton buffer (40 mM Tris pH 8.0, 300 mM NaCl, 1% TX-100), Cholate buffer (40 mM Tris pH 8.0, 300 mM NaCl, 50 mM cholate), and Imidazole Wash Buffer (40 mM Tris pH 8.0, 300 mM NaCl, 50 mM imidazole). MSP1E1 was eluted with three CVs of Elution Buffer (40 mM Tris pH 8.0, 300 mM NaCl, 750 mM imidazole). The eluate was filtered (Millipore; 0.22 μm) and applied to a SEC column (ENC70; BioRad) equilibrated in 20 mM HEPES pH 7.4, 150 mM NaCl, and 1 mM EDTA by fast protein liquid chromatography (FPLC) (NGC system; BioRad). Peak fractions were monitored by UV_280_, pooled, and concentrated to 400–600 μM using a centrifugal device. Final protein concentration was determined by ultraviolet (UV) absorbance at 280 nm. Samples were aliquoted, flash frozen in liquid nitrogen, and stored at −86 °C for up to several months.

### Cx46/50 purification and nanodisc reconstitution

Native Cx46/50 intercellular channels were isolated from native lens fiber cells from sheep^16^. Briefly, lamb eyes were obtained from the Wolverine Packers slaughterhouse (Detroit, MI) and the lenses were removed using a surgical blade and stored at −86 °C. Gap junction intercellular channels were isolated from the core lens fiber tissue, containing C-terminal truncation variants of Cx46 and Cx50 (a.k.a. MP38)^56–59^. Details of the purification procedure are provided below.

Lenses were thawed from −86 °C, core lens fiber cell tissue was dissected from the outer cortical tissue using a surgical blade, and stripped core membranes were prepared as described^60–62^. Total protein concentration was determined by BCA (Pierce) and membranes were stored at −86 °C, in storage buffer (10 mM Tris pH 8.0, 2 mM EDTA, 2 mM EGTA) at a total protein concentration of ~2 mg mL^−1^. Stripped membranes were thawed from −86 °C and solubilized with 10 mM Tris pH 8.0, 2 mM EDTA, 2 mM EGTA, and 1% (wt vol^−1^) *n*-decyl-β-d-maltoside (DM) for 30 min at 37 °C. Insoluble material was cleared by ultra-centrifugation at 146,550 × *g* for 30 minutes at 4 °C. The supernatant was filtered (Millipore; 0.22 μm) and separated by anion-exchange chromatography (UnoQ, BioRad) with buffer A (10 mM Tris pH 8.0, 2 mM EDTA, 2 mM EGTA, 0.3% DM (wt vol^−1^)). Protein was eluted with a 20 CV gradient of buffer B that additionally contained 500 mM NaCl. Elution peaks containing Cx46/50, as determined by SDS-polyacrylamide gel electrophoresis (PAGE), were pooled and applied to a SEC column (Superose 6 Increase 10/300 GL; GE Healthcare) equilibrated with SEC buffer (20 mM HEPES pH 7.4, 150 mM NaCl, 2 mM EDTA, 2 mM EGTA, and 0.3% DM (wt vol^−1^)). Peak fractions containing purified Cx46/50 were pooled and concentrated to 5–6 mg mL^−1^ with a centrifugal device (Vivaspin 6; 50 kDa cut-off filter; Sartorius). Protein concentration was determined by UV absorbance at 280 nm. All chromatography steps were performed by FPLC at 4 °C.

Freshly purified Cx46/50 was reconstituted into MSP1E1 nanodiscs using DMPC lipids, following established procedures^55,63^. Chloroform-solubilized DMPC (Avanti) was dried under nitrogen gas and left under vacuum overnight to remove residual solvent. The resulting thin film was resuspended in 5% DM (wt vol^−1^) to a final DMPC concentration of 30 mM and solubilized in a sonicator bath at 37 °C. DM-solubilized Cx46/50 (5–6 mg mL^−1^) was combined with DMPC at a molar ratio of 0.6:90 (Cx46/50:DMPC) and incubated at 25 °C with gentle agitation for 60 min. Purified MSP1E1 was then added at a final molar ratio 0.6:1:90 (Cx46/50 : MSP1E1 : DMPC) and allowed to incubate at 25 °C for an additional 20 min. Detergent was removed with SM-2 Bio-Beads (BioRad) at a ratio of 30:1 beads : detergent (wt wt^−1^) by overnight incubation at 25 °C with gentle agitation. Bio-Beads were removed by filtration and the sample was ultra-centrifuged at 146,550 × *g* for 15 min at 4 °C to remove insoluble material. The supernatant was filtered (Millipore; 0.22 μm) and applied to an SEC column (Superose 6 Increase 10/300 GL; GE Healthcare) equilibrated in 20 mM HEPES (pH 7.4) and 150 mM NaCl, to separate empty nanodiscs from Cx46/50-embedded nanodiscs. Peak fractions containing both Cx46/50 and MSP1E1, as determined by SDS-PAGE, were collected and concentrated using a centrifugal device (Vivaspin 6; 50 kDa cut-off filter; Sartorius) to a final concentration ~2.5 mg mL^−1^, as determined by UV absorbance at 280 nm (Supplementary Fig. 1a). All chromatography steps were performed by FPLC at 4 °C. The presence of both Cx46 and Cx50 in the final sample was confirmed by western blot analysis using polyclonal antibodies directed against the N-terminal domain of Cx46 (AP11570PU-N, Acris) and the N-terminal domain of Cx50 (LS-C116220, LSBio) (Supplementary Fig. 1b).

### Negative-stain electron microscopy

Cx46/50 lipid nanodisc complexes were prepared for negative-stain EM as described^16^. Briefly, a 3 μl drop of sample (~0.02 mg mL^−1^) was applied to a glow-discharged continuous carbon coated EM specimen grid (Ted Pella), blotted with filter paper and washed two times with detergent-free SEC buffer. The specimen was then stained with freshly prepared 0.75% (wt vol^−1^) uranyl formate (SPI-Chem).

Negatively stained specimens were visualized on a 120 kV TEM (iCorr, Thermo Fisher Scientific) at ×49,000 magnification at the specimen level (Supplementary Fig. 1c). A total of 76 digital micrographs were collected on a 2k × 2k charge-coupled device camera (Eagle 2 K TEM CCD, Thermo Fisher Scientific) with a calibrated pixel size of 4.37 Å and with nominal defocus values ranging from 1.5–3.0 μm. All negative-stain image processing was performed in EMAN2.2^64,65^. After contrast transfer function (CTF) parameters were determined, micrographs with significant astigmatism or drift were excluded based on visual inspection of Thon rings in the power spectrum. Hand-picked particles (7598) were extracted with 84 × 84 pixel box size and subjected to multiple rounds of reference-free two-dimensional (2D) classification, resulting in a final dataset of 3826 “good” particles. Representative class averages are shown in Supplementary Fig. 1c, displaying dimensions consistent with the expectation that Cx46/50 intercellular channels had been reconstituted into a pair of lipid nanodiscs.

### CryoEM specimen preparation and data collection

Samples were prepared for CryoEM by applying 5 μl freshly purified Cx46/50 lipid nanodisc complex (~2.5 mg mL^−1^) to a glow-discharged holey carbon grid (Quantifoil R 1.2/1.3, 400 mesh) for 10 s. The grid was blotted for 4.0 s and plunge frozen in liquid ethane using a Vitrobot Mark IV (Thermo Fisher Scientific) at 100% humidity and stored under liquid nitrogen.

CryoEM specimen grids were imaged on a Titan Krios (Thermo Fisher Scientific) operated at 300 kV. Dose-fractionated image stacks were recorded on a Falcon 3EC Direct Electron Detector (Thermo Fisher Scientific) at ×120,000 nominal magnification in counting mode, with a calibrated pixel size of 0.649 Å pixel^−1^ (Supplementary Fig. 2a). The dose rate was 1.14 e^−^ pixel^−1^ s^−1^, with five frames per second collected for a total exposure of 30 s, resulting in a total dose for each exposure of ~52.5 e^−^ Å^−2^. A dataset of 2087 movies was obtained with nominal defocus values ranging from 1.0–2.2 μm and data collection parameters were controlled in an automated manner using EPU (Thermo Fisher Scientific).

### CryoEM image processing for high-resolution workflow

The full dataset of 2087 movies were corrected for beam-induced motion in RELION-3.0^66^ and CTF estimation was performed with Gctf^67^ on the non-dose-weighted, aligned micrographs. Laplacian-of-Gaussian autopicking in RELION-3.0 yielded an initial set of 756,374 picks, which after multiple rounds of 2D classification left 183,784 bona fide particles (binned to a 64 pixel box, 3.894 Å pixel^−1^). These particles were used to generate a de novo initial model in RELION and subsequent 3D refinement of these particles yielded a map at 8.0 Å resolution (64 pixel box, 3.894 Å pixel^−1^). This map was low-pass-filtered to 20 Å and projected in 14 unique orientations to perform 3D template-based autopicking in RELION-3.0 to yield 1,210,797 particle picks. Following multiple rounds of 2D classification, this dataset yielded 379,423 “good” particles (200-pixel box, 1.947 Å pixel^−1^) (Supplementary Fig. 2b). Particles that had been translated within 20 Å of their nearest neighbor were removed to prevent invalidation of gold-standard FSC by duplicate particles. Removal of 120,228 duplicates yielded a 259,195 refined particle set.

This particle set was then re-extracted (1.62 Å pixel^−1^, 280-pixel box) and subjected to 3D refinement (D6 symmetry), yielding a map at 3.3 Å resolution. A subsequent round of de-duplication (20 Å cut-off) yielded 227,618 particles that were again re-extracted (0.974 Å pixel^−1^, 512-pixel box) and subjected to 3D refinement (D6 symmetry), which improved the resolution to 3.2 Å. Two rounds of Bayesian polishing and CTF refinement (per-particle defocus, per-micrograph astigmatism) with subsequent 3D refinement (D6 symmetry) yielded a map at 2.7 Å resolution. Particles were then completely unbinned (400-pixel box, 0.649 Å pixel^−1^) and subjected to another round of 3D refinement (D6 symmetry), yielding a map that reached the same resolution prior to unbinning (2.7 Å). Bayesian polishing and subsequent 3D refinement of these particles showed no significant improvement. The maps generated up to this stage were obtained without applied masks and there was no evidence of resolved lipids within the intracellular leaflet (see Supplementary Fig. 2, Step 2).

At this stage, the newly developed tools in RELION-3.1-beta^68^ were implemented to estimate the degree of beam tilt and high-order aberrations (threefold and fourfold astigmatism) present in the particle images. Subsequent 3D refinement (D6 symmetry) improved the resolution to 2.2 Å. Particles that had been translated to within 35 Å of their nearest neighbor (6224 particles) were again removed to prevent invalidation of the gold-standard FSC from duplicate particles. The remaining 221,394 particles were subjected to 3D classification into 2 classes with D6 symmetry and a tight solvent mask. Approximately 89% of the particles (196,320) fell into one class that was subsequently refined to 2.2 Å resolution (D6 symmetry and solvent mask applied). The remaining 11% of particles (26,005) yielded a 2.0 Å resolution map after 3D refinement (D6 symmetry and solvent mask applied). There was no clear difference in protein conformation between these two classes; however, the high-resolution class was characterized by a particle set with comparatively lower defocus values (mean defocus = 1.20 ± 0.29 μm (SD)) as compared to the lower resolution class (mean defocus = 1.86 ± 0.52 μm (SD)) (https://github.com/huwjenkins). All subsequent processing steps were performed on this high-resolution particle set.

Particles were re-extracted with an expanded box size (initially to 448 pixels) to mitigate delocalized CTF signal from particle images with relatively high defocus. New polishing parameters were obtained by running the Bayesian polishing job type in RELION-3.1-beta in “Training mode” on a random 5000 particle subset of these refined particles. Bayesian polishing was performed with these new parameters and the subsequent 3D refinement (D6 symmetry and solvent mask applied) improved the resolution slightly to 1.97 Å. This process was iterated multiple times with successive increase in box size and incrementally tighter solvent mask applied during Bayesian polishing until no further improvements were observed, resulting in a final box size of 768 pixels. The final map refined to a global 1.90 Å resolution with D6 symmetry and 2.3 Å resolution without symmetry (gold standard, 0.143 cut-off)^69^ (Supplementary Figs. 2c and 3a). The asymmetric reconstruction showed no discernable difference in protein conformation as compared to the D6-symmeterized map. Local resolution of the final map was estimated in RELION-3.1-beta^68^ and local resolution-filtered maps were generated for model building (Supplementary Figs. 2d and 3b). A schematic illustrating this high-resolution CryoEM workflow is presented in (Supplementary Fig. 2c).

### CryoEM image processing workflow for lipid classification

For classification and analysis of lipid configurational/conformational heterogeneity, a modified workflow starting from the totally unbinned 227,618 particle set (0.649 Å pixel^−1^, 400-pixel box), which yielded the 2.7 Å resolution map, was applied, as described here (and illustrated in Supplementary Fig. 4a). The particle set was subjected to 3D classification (eight classes), with D6 symmetry and a generous solvent mask applied. Two of the eight classes yielded maps in which the lipid configuration was unambiguously resolved: assigned as PC Class 1 containing 9190 particles (~4% of the data) and PC Class 3 containing 6944 particles (~3% of the data). Overlapping configurations were resolved in two of the other 3D classes, and so particles from these classes were combined and subjected to a second round of 3D classification with only 2 classes and a tight solvent mask applied. This yielded one class with unresolved lipid configurations and a second class in which the lipid configuration was unambiguously resolved: assigned PC Class 2 containing 6075 particles (~3% of the data). Particles assigned to PC Class 1, 2, and 3 were separately subjected to a final round of 3D refinement with a solvent mask and D6 symmetry applied (Supplementary Fig. 4a). The final reconstructions from particles in each of these classes all reached ~2.5 Å resolution (gold standard, 0.143 cut-off) (Supplementary Fig. 4b). Local resolution was estimated in RELION-3.1-beta and local resolution-filtered maps were generated for model building (Supplementary Fig. 4c).

We note that alternative approaches to heterogeneity analysis were also pursued. For example, using the signal-subtraction and symmetry expansion methods in attempt to characterize the specimen heterogeneity at the single subunit level^70^. However, these approaches did not resolve any additional PC lipid conformations/configurations than those captured by the approach described above. It also did not help to resolve the Cx46 and Cx50 isoform heterogeneity.

### Atomic modeling, refinement, and validation

For all atomic models of Cx46 and Cx50, initial models were derived from previously reported CryoEM structure of amphipol-stabilized Cx46 and Cx50 (PDB 6MHQ and 6MHY^16^, respectively). Initial models were fit as rigid bodies into the D6-symmetrized CryoEM maps with applied local resolution-filtering using UCSF Chimera^71^. All atom models for Cx46 and Cx50 were further built into the CryoEM density maps with COOT^72^ and subjected to real-space refinement in PHENIX^73^ with secondary structure and non-crystallographic symmetry (D6) restraints applied. Several iterations of manual adjustment of the protein model in COOT, followed by real-space refinement in PHENIX, were performed while monitoring model quality with MolProbity^74^ and quality of side chain fit with EMRinger^75^. Coordinate and restraint files for the DMPC ligands were generated with PHENIX eLBOW^76^. DMPC molecules were manually fit into the CryoEM density with COOT. Since density for the PC head groups was not resolved in the high-resolution ensemble CryoEM map (1.9 Å map), head group, and acyl-chain atoms that could not be accommodated by the density were deleted. For the PC lipid classes 1–3, the postprocessed maps from RELION were low-pass-filtered to 3.5 Å resolution, to facilitate modeling of the fully resolved PC lipids. COOT was further used to manually place water molecules into solvent densities of the CryoEM maps. Appropriate placement of waters was determined by the following three criteria: (1) confirmation of at least two hydrogen bond donor/acceptor interactions with the FindHBond tool in UCSF Chimera (<4 Å donor–acceptor distance) and by visual inspection, (2) confirmation of solvent densities consistently observed in both gold-standard separated half-maps (contoured ≥ 2.5*σ*), and (3) as an additional measure we looked for density overlap between the local resolution-filtered CryoEM map (contoured ≥ 5.3*σ*) and the time-averaged water density map generated by equilibrium MD simulation (contoured ≥ 5.0*σ*) to help assign weak experimental water densities (Supplementary Fig. 8, see calculation of water density maps from MD described in “Methods” below). However, not all of the assigned CryoEM water densities were observed by MD (76% of waters were observed at equivalent positions by CryoEM and MD). Several iterations of real-space refinement on the entire model were completed until refinement statistics converged.

### Disclosure of unresolved heteromeric assemblies of Cx46/50

All models of Cx46 and Cx50 were built using D6 (12-fold) symmetrized CryoEM maps. As native Cx46/50 intercellular channels may form homomeric and/or various patterns of heteromeric/heterotypic configurations^16,77,78^, this map most likely represent a heterogeneous mixture of these two isoforms^16^. This approach was chosen, because all attempts to separate the heteromeric/heterotypic assembly of these two isoforms using image classification procedures were unsuccessful (presumably due to the close sequence and structural similarity of these two isoforms) (see also ref. ^16^). Indeed, Cx46 and Cx50 are 80% identical and 89% similar in sequence over the resolved structural domains, whereas sites of difference are typically at solvent-exposed regions (Supplementary Fig. 6a). Despite this limitation, all atomic models generated by this approach showed good stereochemical refinement statistics (see Supplementary Table 1) and significant improvements to the previously described amphipol-stabilized models that were refined to 3.4 Å resolution (Supplementary Fig. 5a-e). It is important to note that sites in the density maps where the sequence of Cx46 and Cx50 are identical or similar, both models fit well into the D6-symmetrized map and these regions tend to display well-resolved side chain density (Supplementary Fig. 3c, d). Over regions where the sequence of Cx46 and Cx50 differ, side chain density is sometimes weaker and/or displays appearance of density consistent with a mixture of both isoforms (Supplementary Fig. 3c, d). These observations are possibly due to the imposed D6 symmetry averaging of density belonging to two different sidechains in these areas or relative flexibility at these sites, as many of these residues are also solvent exposed. In these areas of difference, where EM density is observed, both Cx46 and Cx50 can be fit into the density equally well (Supplementary Fig. 3c, d). Nevertheless, caution should be used with interpretation of the conformational details at these sites of isoform difference.

### Molecular dynamics simulations

Visual MD (VMD) v1.9.3^79^ was used to build systems for sheep Cx46 and Cx50 in a dual lipid bilayer with varying salt conditions, designed to mimic either the cellular environment (cytoplasmic KCl, extracellular NaCl) or experimental CryoEM conditions (uniform NaCl). To produce unbiased analysis of water and lipid interactions, all water and lipid molecules derived by CryoEM analysis were removed from the Cx46 and Cx50 models prior to the MD setup. Each system comprised the full dodecameric gap junction intercellular channel, prepared in explicit water (model TIP3P) and embedded in two lipid bilayers composed of DMPC, mimicking the cell-to-cell junction. For all models, sidechains were protonated according to neutral conditions and the HSD model was used for all histidine residues. Disulfide bonds identified in the experimental structures were enforced. Amino acids corresponding to the ICL (residues 110–136 in sheep Cx46 and residues 110–148 in sheep Cx50) and CTD (residues 225–413 in sheep Cx46 and residues 237–440 in sheep Cx50) were not included for the MD simulations, as experimental data describing the structure of these large flexible domains (~30 residue ICL and ~200 residue CTD in Cx46 and Cx50) are missing. The introduced N- and C-terminal residues resulting from the missing ICL segment (sheep Cx46 R109 and K137; sheep Cx50 R109 and R149) were neutralized. All of the systems were modified with an N-terminal acetylation (at the starting residue Gly 2) in VMD through an all-atom acetylation patch in the automated PSF-Builder, in accordance with previously described proteomics analysis on native Cx46/50^16,59,80^ and the expectation that this species would predominate in cells^81^. A complete list of modeled residues for each system is provided in Supplementary Fig. 7a.

The prepared protein structures were submerged in a hydration shell using Solvate 1.0.1^82^. Water was removed from sections of the channel corresponding to transmembrane domains, based on hydrophobic character and localization of lipid nanodisc observed in the experimental CryoEM data (±20–50 Å from the center of the channel). The CHARMM-GUI membrane builder^83^ was used to build the DMPC bilayers (pre-melted), with dimensions of 154 × 154 Å for Cx46 and Cx50, and lipids overlapping with protein were removed. The entire system was then placed in a water box with dimensions 147 × 147 × 174 Å for both Cx46 and Cx50, using VMD’s Solvate plugin. The system was neutralized using the Autoionize plugin, then 150 mM KCl and 150 mM NaCl was added to the solvent areas corresponding to intracellular and extracellular regions of the simulation box for the “KCl” systems, whereas the “NaCl” systems contained 150 mM NaCl for the entire box. A summary of atoms counts for each system is provided in Supplementary Fig. 7a.

Graphics processing unit (GPU)-accelerated nanoscale MD (NAMD) 2.13^84^ was used for all classical MD simulations, using the CHARMM36 force field^85^ for all atoms and TIP3P explicit model for water. Each system was prepared following the same minimization and equilibration protocol as follows. An initial minimization step, where the lipids, solvent, and ions were allowed to minimize around the protein, was performed, with the protein harmonically constrained for 1 ns, with 1 fs timestep and constant number of particles, volume and temperature (310 K) (NVT ensemble). A second minimization step was applied, where the system was free to minimize with a harmonic constraint on the protein backbone to ensure stable quaternary structure for 1 ns—lipids relax and compress during minimization steps with minimized dimensions equal to the water box (14.7 × 14.7). The entire system was then released from restraints and subjected to all-atom equilibration runs employing Langevin thermostat, with a constant number of particles, pressure (1 atm) and temperature (310 K) (NPT ensemble), with 2 fs time steps and allowed to proceed for 30 ns. Periodic boundary conditions were used to allow for the smoothed particle mesh Ewald calculation of electrostatics. Finally, two independent 100 ns production runs were seeded with randomly initialized velocities from the initial equilibration simulation—providing 200 ns of production for each system. r.m.s.d. and r.m.s.f. were calculated using VMD, and r.m.s.f. values were displayed to the protein structure using UCSF Chimera (Supplementary Fig. 7b-d). All systems approached a steady r.m.s.d. within 30 ns of the equilibration phase (Supplementary Fig. 7b) and r.m.s.f. values appeared well-behaved over the production periods, including regions corresponding to the NTH domain^16^ (Supplementary Fig. 7c, d). The only significant fluctuations (i.e., >2.5 Å) occurred at the TM2, TM3, and TM4 cytoplasmic termini, which is expected, as these regions form the boundary to the intrinsically disordered ICL and CTD regions of the protein (not modeled). All systems maintained an electro-chemical seal to extracellular sodium ions (Na^+^) around the extracellular docking domains during MD simulation.

### Calculation of MD-based density maps

The Volmap plugin in VMD was used for the calculation of volumetric density maps, by replacing each atom with a normalized Gaussian distribution, whose standard deviation is equal to the radius of the atom. All of the Gaussians are summed and distributed on a grid for each frame of the simulation. The grids were re-sampled to a final voxel resolution of 0.649 Å to match the pixel size used in the CryoEM reconstruction. Water, ion, and lipid maps were calculated from each of two 100 ns production runs and subsequently averaged and symmetrized (D6 symmetry) with the relion_image_handler tool in RELION-3.0^66^. Lipid and water density maps produced from Cx46 and Cx50 MD simulations contained significant overlap to each other and to the CryoEM maps; however, the maps produced from Cx50 MD simulations were of higher quality and were selected for detailed comparative analysis to the CryoEM density maps (Fig. 3 and Supplementary Fig. 8). Ion density maps showed only a few features and did not correspond to densities observed by CryoEM and were therefore excluded from further analysis.

### Area per lipid and lipid order parameter calculations

Area per lipid for each membrane, separated by intracellular and extracellular leaflet, were calculated using the program FATSLiM^86^, and were used as an indicator of equilibration of the lipid systems (Supplementary Fig. 9a).

1 $${{S}}_{{\mathrm{CD}}} \equiv - \left\langle {\frac{{3\cos ^2\left( {\theta _{{\mathrm{CD}}}} \right) - 1}}{2}} \right\rangle$$

The *S*_CD_ lipid order parameter, as defined by Eq. 1, measures the orientation of the SN1 and SN2 acyl chains by monitoring the angle that each acyl C–H vector makes with the bilayer normal *θ*_CD_. The calculations of *S*_CD_ were done using the VMD script *calc_op.tcl*^87^. To analyze the distance dependence of *S*_CD_ in the respective membrane leaflets, the averaged *S*_CD_ values were calculated in 5 Å concentric shells around the protein (SN1 and SN2 calculated, separately). *S*_CD_ of lipids from both membranes are averaged together, whereas the intra- and extracellular leaflets were averaged separately (Supplementary Fig. 9b-e). To visualize the order parameter mapped to the structure, the time-averaged *S*_CD_ values were calculated for each lipid (SN1 and SN2 combined values for acyl carbons 4–11) and colored according to this value using UCSF Chimera (Fig. 3c).

### MD lipid configuration analysis

Analysis of PC lipid configuration (i.e., acyl-chain positioning) was performed using in-house scripts to assess how phospholipids are organized within the extracellular leaflet of the Cx46 and Cx50 intercellular channels during MD simulation, as compared to the PC configurations classified by CryoEM. This was done by counting the instances of a single DMPC molecule occupying the region bounded by the MD-based lipid density, contoured at *σ*_min_ = 8 (Fig. 3a). The lipid acyl-chain density maps calculated from the Cx50 MD simulations reveal more than 19 resolved rods (*i*) of density per connexin subunit (12 subunits) and each rod was arbitrarily numbered 1 through 19 (total of 228 acyl-chain positions). A lipid was classified in a state when both acyl chains occupied a density, state ≡ “*i* − *j*” (where *i* ≠ *j*). A rod density is considered occupied if at least five carbons of a lipid’s acyl chain are within the density, such that *σ*_carbon_ ≥ *σ*_min_. This classification scheme was applied to every lipid within 15 Å of the protein, over each frame (0.1 ns per frame). To analyze the dynamics of lipids surrounding the protein, the state of each lipid (e.g., “1–2,” “1–5,” “none,” etc.) was monitored and recorded at every frame providing a time series of lipid configurational dynamics in state space (Fig. 4d).

### Statistical analysis

Ninety-five percent confidence intervals for Cα r.m.s.f. values are reported (*n* = 24) using a two-tailed Student’s *t*-test. FSC was performed using gold standard methods with a 0.143 cut-off criteria^69^. No statistical methods were used to predetermine sample size for the CryoEM dataset. The experiments were not randomized and investigators were not blinded to allocation during experiments and outcome assessment.

### Figures and movie preparation

Figure panels and movies were created using Chimera^71^, ChimeraX^88^, VMD^79^, and Blender^89^. Figures were composited in Adobe Photoshop. Movies were composited in Blender.

### Reporting summary

Further information on research design is available in the Nature Research Reporting Summary linked to this article.

## Supplementary information

Supplementary Information

Supplementary Movie 1

Supplementary Movie 2

Supplementary Movie 3

Supplementary Movie 4

Supplementary Movie 5

Supplementary Movie 6

Reporting Summary

## Data Availability

Data supporting the findings of this study are available from the corresponding author upon reasonable request. A reporting summary for this Article is available as a Supplementary Information file. CryoEM density maps, including half-maps, pre- and postprocessed maps, and masks have been deposited to the Electron Microscopy Data Bank (EMD-22358, EMD-22382, EMD-22390, and EMD-22391). Coordinates for Cx50 and Cx46 atomic models have been deposited to the Protein Data Bank: PDB7JJP and PDB7JKC correspond to the high-resolution models, PDB7JLW and PDB7JMD correspond to the models from PC Class 1, PDB7JM9 and PDB7JN0 correspond to PC Class 2, and PDB7JMC and PDB7JN1 correspond to PC Class 3. The original multi-frame micrographs have been deposited to EMPIAR (EMPIAR-10480). MD trajectory files and MD-based density maps have been deposited to Zenodo (10.5281/zenodo.3951861).

## Code availability

In-house code developed for the analysis of MD trajectories to assess lipid configurational state dynamics have been deposited on the Reichow Lab GitHub repository (https://github.com/reichow-lab/Flores-Haddad-Dolan_et-al.NatCommun2020) and Zenodo (10.5281/zenodo.3955131).

## References

[1] Goodenough DA, Paul DL (2009). Gap junctions. Cold Spring Harb. Perspect. Biol..

[2] Sohl G, Willecke K (2004). Gap junctions and the connexin protein family. Cardiovasc. Res..

[3] Harris AL (2007). Connexin channel permeability to cytoplasmic molecules. Prog. Biophys. Mol. Biol..

[4] Bonacquisti EE, Nguyen J (2019). Connexin 43 (Cx43) in cancer: Implications for therapeutic approaches via gap junctions. Cancer Lett..

[5] Delmar, M. et al. Connexins and disease. *Cold Spring Harb. Perspect. Biol.*10.1101/cshperspect.a029348 (2017). 10.1101/cshperspect.a029348 PMC6120696 28778872

[6] Garcia IE (2016). Connexinopathies: a structural and functional glimpse. BMC Cell Biol..

[7] Aasen T, Mesnil M, Naus CC, Lampe PD, Laird DW (2016). Gap junctions and cancer: communicating for 50 years. Nat. Rev. Cancer.

[8] Makowski L, Caspar DL, Phillips WC, Goodenough DA (1977). Gap junction structures. II. Analysis of the X-ray diffraction data. J. Cell Biol..

[9] Revel JP, Karnovsky MJ (1967). Hexagonal array of subunits in intercellular junctions of the mouse heart and liver. J. Cell Biol..

[10] Sosinsky GE, Nicholson BJ (2005). Structural organization of gap junction channels. Biochim. Biophys. Acta.

[11] Malewicz B, Kumar VV, Johnson RG, Baumann WJ (1990). Lipids in gap junction assembly and function. Lipids.

[12] Cascio M (2005). Connexins and their environment: effects of lipids composition on ion channels. Biochim. Biophys. Acta.

[13] Puebla C, Retamal MA, Acuna R, Saez JC (2017). Regulation of connexin-based channels by fatty acids. Front. Physiol..

[14] Kistler J, Goldie K, Donaldson P, Engel A (1994). Reconstitution of native-type noncrystalline lens fiber gap junctions from isolated hemichannels. J. Cell Biol..

[15] Locke D, Harris AL (2009). Connexin channels and phospholipids: association and modulation. BMC Biol..

[16] Myers JB (2018). Structure of native lens connexin 46/50 intercellular channels by cryo-EM. Nature.

[17] Denisov IG, Grinkova YV, Lazarides AA, Sligar SG (2004). Directed self-assembly of monodisperse phospholipid bilayer nanodiscs with controlled size. J. Am. Chem. Soc..

[18] Maeda S (2009). Structure of the connexin 26 gap junction channel at 3.5 A resolution. Nature.

[19] Bennett BC (2016). An electrostatic mechanism for Ca(2+)-mediated regulation of gap junction channels. Nat. Commun..

[20] Gong XQ, Nicholson BJ (2001). Size selectivity between gap junction channels composed of different connexins. Cell Commun. Adhes..

[21] Trexler EB, Bukauskas FF, Kronengold J, Bargiello TA, Verselis VK (2000). The first extracellular loop domain is a major determinant of charge selectivity in connexin46 channels. Biophys. J..

[22] Kronengold J, Trexler EB, Bukauskas FF, Bargiello TA, Verselis VK (2003). Pore-lining residues identified by single channel SCAM studies in Cx46 hemichannels. Cell Commun. Adhes..

[23] Verselis VK, Trelles MP, Rubinos C, Bargiello TA, Srinivas M (2009). Loop gating of connexin hemichannels involves movement of pore-lining residues in the first extracellular loop domain. J. Biol. Chem..

[24] Oh S, Verselis VK, Bargiello TA (2008). Charges dispersed over the permeation pathway determine the charge selectivity and conductance of a Cx32 chimeric hemichannel. J. Physiol..

[25] Kwon T (2012). Molecular dynamics simulations of the Cx26 hemichannel: insights into voltage-dependent loop-gating. Biophys. J..

[26] Kwon T, Harris AL, Rossi A, Bargiello TA (2011). Molecular dynamics simulations of the Cx26 hemichannel: evaluation of structural models with Brownian dynamics. J. Gen. Physiol..

[27] Zonta F, Polles G, Zanotti G, Mammano F (2012). Permeation pathway of homomeric connexin 26 and connexin 30 channels investigated by molecular dynamics. J. Biomol. Struct. Dyn..

[28] Bargiello TA, Tang Q, Oh S, Kwon T (2012). Voltage-dependent conformational changes in connexin channels. Biochim. Biophys. Acta.

[29] Tong X (2015). The first extracellular domain plays an important role in unitary channel conductance of Cx50 gap junction channels. PLoS ONE.

[30] Lopez W (2016). Mechanism of gating by calcium in connexin hemichannels. Proc. Natl Acad. Sci. USA.

[31] Garcia IE (2018). The syndromic deafness mutation G12R impairs fast and slow gating in Cx26 hemichannels. J. Gen. Physiol..

[32] Rubinos C, Sanchez HA, Verselis VK, Srinivas M (2012). Mechanism of inhibition of connexin channels by the quinine derivative N-benzylquininium. J. Gen. Physiol..

[33] Banks EA (2009). Connexin mutation that causes dominant congenital cataracts inhibits gap junctions, but not hemichannels, in a dominant negative manner. J. Cell Sci..

[34] Berthoud VM (2013). Connexin50D47A decreases levels of fiber cell connexins and impairs lens fiber cell differentiation. Invest. Ophthalmol. Vis. Sci..

[35] Reis LM (2013). Whole exome sequencing in dominant cataract identifies a new causative factor, CRYBA2, and a variety of novel alleles in known genes. Hum. Genet..

[36] White TW, Bruzzone R, Wolfram S, Paul DL, Goodenough DA (1994). Selective interactions among the multiple connexin proteins expressed in the vertebrate lens: the second extracellular domain is a determinant of compatibility between connexins. J. Cell Biol..

[37] White TW, Paul DL, Goodenough DA, Bruzzone R (1995). Functional analysis of selective interactions among rodent connexins. Mol. Biol. Cell.

[38] Nakagawa S (2011). Asparagine 175 of connexin32 is a critical residue for docking and forming functional heterotypic gap junction channels with connexin26. J. Biol. Chem..

[39] Cottrell GT, Burt JM (2005). Functional consequences of heterogeneous gap junction channel formation and its influence in health and disease. Biochim. Biophys. Acta.

[40] Bai D, Wang AH (2014). Extracellular domains play different roles in gap junction formation and docking compatibility. Biochem. J..

[41] Schadzek P (2016). The cataract related mutation N188T in human connexin46 (hCx46) revealed a critical role for residue N188 in the docking process of gap junction channels. Biochim. Biophys. Acta.

[42] Silander K (1998). Spectrum of mutations in Finnish patients with Charcot-Marie-Tooth disease and related neuropathies. Hum. Mutat..

[43] Primignani P (2003). A novel dominant missense mutation–D179N–in the GJB2 gene (Connexin 26) associated with non-syndromic hearing loss. Clin. Genet..

[44] Deeley JM (2008). Human lens lipids differ markedly from those of commonly used experimental animals. Biochim. Biophys. Acta.

[45] Lampe PD (1991). In vitro assembly of gap junctions. J. Struct. Biol..

[46] Mabrey S, Sturtevant JM (1976). Investigation of phase transitions of lipids and lipid mixtures by sensitivity differential scanning calorimetry. Proc. Natl Acad. Sci. USA.

[47] Shaw AW, McLean MA, Sligar SG (2004). Phospholipid phase transitions in homogeneous nanometer scale bilayer discs. FEBS Lett..

[48] Vermeer LS, de Groot BL, Reat V, Milon A, Czaplicki J (2007). Acyl chain order parameter profiles in phospholipid bilayers: computation from molecular dynamics simulations and comparison with 2H NMR experiments. Eur. Biophys. J..

[49] Khakbaz P, Klauda JB (2018). Investigation of phase transitions of saturated phosphocholine lipid bilayers via molecular dynamics simulations. Biochim. Biophys. Acta Biomembr..

[50] Caspar DL, Goodenough DA, Makowski L, Phillips WC (1977). Gap junction structures. I. Correlated electron microscopy and x-ray diffraction. J. Cell Biol..

[51] Schubert AL, Schubert W, Spray DC, Lisanti MP (2002). Connexin family members target to lipid raft domains and interact with caveolin-1. Biochemistry.

[52] Locke D, Liu J, Harris AL (2005). Lipid rafts prepared by different methods contain different connexin channels, but gap junctions are not lipid rafts. Biochemistry.

[53] Hunte C (2005). Specific protein-lipid interactions in membrane proteins. Biochem. Soc. Trans..

[54] Spray DC, Rozental R, Srinivas M (2002). Prospects for rational development of pharmacological gap junction channel blockers. Curr. Drug Targets.

[55] Ritchie TK (2009). Chapter 11 - Reconstitution of membrane proteins in phospholipid bilayer nanodiscs. Methods Enzymol..

[56] Kistler J, Christie D, Bullivant S (1988). Homologies between gap junction proteins in lens, heart and liver. Nature.

[57] Kistler J, Schaller J, Sigrist H (1990). MP38 contains the membrane-embedded domain of the lens fiber gap junction protein MP70. J. Biol. Chem..

[58] White TW, Bruzzone R, Goodenough DA, Paul DL (1992). Mouse Cx50, a functional member of the connexin family of gap junction proteins, is the lens fiber protein MP70. Mol. Biol. Cell.

[59] Wang Z, Schey KL (2009). Phosphorylation and truncation sites of bovine lens connexin 46 and connexin 50. Exp. Eye Res..

[60] Reichow SL (2013). Allosteric mechanism of water-channel gating by Ca2+-calmodulin. Nat. Struct. Mol. Biol..

[61] Gold MG (2012). AKAP2 anchors PKA with aquaporin-0 to support ocular lens transparency. EMBO Mol. Med..

[62] Reichow SL, Gonen T (2008). Noncanonical binding of calmodulin to aquaporin-0: implications for channel regulation. Structure.

[63] Efremov RG, Gatsogiannis C, Raunser S (2017). Lipid nanodiscs as a tool for high-resolution structure determination of membrane proteins by single-particle cryo-EM. Methods Enzymol..

[64] Tang G (2007). EMAN2: an extensible image processing suite for electron microscopy. J. Struct. Biol..

[65] Ludtke SJ (2016). Single-particle refinement and variability analysis in EMAN2.1. Methods Enzymol..

[66] Zivanov, J. et al. New tools for automated high-resolution cryo-EM structure determination in RELION-3. *eLife* **7**, 10.7554/eLife.42166 (2018). 10.7554/eLife.42166 PMC6250425 30412051

[67] Zhang K (2016). Gctf: Real-time CTF determination and correction. J. Struct. Biol..

[68] Zivanov J, Nakane T, Scheres SHW (2020). Estimation of high-order aberrations and anisotropic magnification from cryo-EM data sets in RELION-3.1. IUCrJ.

[69] Scheres SH, Chen S (2012). Prevention of overfitting in cryo-EM structure determination. Nat. Methods.

[70] Scheres SH (2016). Processing of structurally heterogeneous cryo-EM data in RELION. Methods Enzymol..

[71] Pettersen EF (2004). UCSF Chimera-a visualization system for exploratory research and analysis. J. Comput. Chem..

[72] Emsley P, Lohkamp B, Scott WG, Cowtan K (2010). Features and development of Coot. Acta Crystallogr..

[73] Afonine PV (2018). Real-space refinement in PHENIX for cryo-EM and crystallography. Acta Crystallogr. D. Struct. Biol..

[74] Williams CJ (2018). MolProbity: more and better reference data for improved all-atom structure validation. Protein Sci..

[75] Barad BA (2015). EMRinger: side chain-directed model and map validation for 3D cryo-electron microscopy. Nat. Methods.

[76] Moriarty NW, Grosse-Kunstleve RW, Adams PD (2009). Electronic Ligand Builder and Optimization Workbench (eLBOW): a tool for ligand coordinate and restraint generation. Acta Crystallogr. D. Biol. Crystallogr..

[77] Konig N, Zampighi GA (1995). Purification of bovine lens cell-to-cell channels composed of connexin44 and connexin50. J. Cell Sci..

[78] Jiang JX, Goodenough DA (1996). Heteromeric connexons in lens gap junction channels. Proc. Natl Acad. Sci. USA.

[79] Humphrey W, Dalke A, Schulten K (1996). VMD: visual molecular dynamics. J. Mol. Graph..

[80] Shearer D, Ens W, Standing K, Valdimarsson G (2008). Posttranslational modifications in lens fiber connexins identified by off-line-HPLC MALDI-quadrupole time-of-flight mass spectrometry. Invest. Ophthalmol. Vis. Sci..

[81] Varland S, Osberg C, Arnesen T (2015). N-terminal modifications of cellular proteins: the enzymes involved, their substrate specificities and biological effects. Proteomics.

[82] Grubmuller H, Heymann B, Tavan P (1996). Ligand binding: molecular mechanics calculation of the streptavidin-biotin rupture force. Science.

[83] Wu EL (2014). CHARMM-GUI Membrane Builder toward realistic biological membrane simulations. J. Comput. Chem..

[84] Phillips JC (2005). Scalable molecular dynamics with NAMD. J. Comput. Chem..

[85] Huang J, MacKerell AD (2013). CHARMM36 all-atom additive protein force field: validation based on comparison to NMR data. J. Comput. Chem..

[86] Buchoux S (2017). FATSLiM: a fast and robust software to analyze MD simulations of membranes. Bioinformatics.

[87] Piggot TJ, Allison JR, Sessions RB, Essex JW (2017). On the calculation of acyl chain order parameters from lipid simulations. J. Chem. Theory Comput..

[88] Goddard TD (2018). UCSF ChimeraX: meeting modern challenges in visualization and analysis. Protein Sci..

[89] Community, B. O. Blender–a 3D modelling and rendering package. http://www.blender.org (Stichting Blender Foundation, 2018).

