# Supplementary material for: Connexin-46/50 in a dynamic lipid environment resolved by CryoEM at 1.9 Å
Source: Nat Commun. 2020 Aug 28;11:4331. doi: 10.1038/s41467-020-18120-5 (PMC7455559; doi:10.1038/s41467-020-18120-5)
Supplement: Supplementary file 1 — Supplementary Information [file 41467_2020_18120_MOESM1_ESM.pdf]

Supplementary Information

Connexin-46/50 in a dynamic lipid environment resolved by CryoEM at 1.9 Å

Authors: Jonathan A. Flores[1,†], Bassam G. Haddad[2,†], Kimberly A. Dolan[2,3,†], Janette B. Myers[2], Craig C. Yoshioka[4], Jeremy Copperman[4], Daniel M. Zuckerman[4] and Steve L. Reichow[1,2,*]

Affiliations:

[1]Department of Chemical Physiology and Biochemistry, Oregon Health and Science University, Portland OR 97239, U.S.A.

[2]Department of Chemistry, Portland State University, Portland OR 97201, U.S.A.

[3]Current Address: Biophysics Graduate Group, University of California, Berkeley, CA 94720, U.S.A.

[4]Department of Biomedical Engineering, Oregon Health and Science University, Portland OR 97239, U.S.A.

[†] Indicates equal contribution

* Correspondence: reichow@pdx.edu (S.L.R.)

Supplementary Tables and Figures

Supplementary Table 1

	Cx46	Cx50	Cx46 *PC Class 1*	Cx50 *PC Class 1*	Cx46 *PC Class 2*	Cx50 *PC Class 2*	Cx46 *PC Class 3*
**Data collection and processing**							
Magnification	120,000	120,000	120,000	120,000	120,000	120,000	120,000
Voltage (kV)	300	300	300	300	300	300	300
Electron exposure (e–/Å^2)	52.5	52.5	52.5	52.5	52.5	52.5	52.5
Defocus range (μm)	-1.0 to -2.2	-1.0 to -2.2	-1.0 to -2.2	-1.0 to -2.2	-1.0 to -2.2	-1.0 to -2.2	-1.0 to -2.2
Pixel size (Å)	0.649	0.649	0.649	0.649	0.649	0.649	0.649
Symmetry imposed	D6	D6	D6	D6	D6	D6	D6
Initial particle images (no.)	1,210,797	1,210,797	1,210,797	1,210,797	1,210,797	1,210,797	1,210,797
Final particle images (no.)	26,005	26,005	6,073	6,073	9,188	9,188	6,942
Map resolution (Å)	1.90	1.90	2.52	2.52	2.47	2.47	2.45
FSC threshold	0.143	0.143	0.143	0.143	0.143	0.143	0.143
Map resolution range (Å)	1.90–3.30	1.90–3.30	2.42–4.56	2.42–4.56	2.33–4.14	2.33–4.14	2.47–4.33
**Refinement**							
Initial model used (PDB code)	6MHQ	6MHY	6MHQ	6MHY	6MHQ	6MHY	6MHQ
Model resolution (Å)	1.99	1.99	2.55	2.52	2.35	2.35	2.63
FSC threshold	0.5	0.5	0.5	0.5	0.5	0.5	0.5
Model resolution range (Å)	-	-	-	-	-	-	-
Map sharpening *B* factor (Å^2)	-25.00	-25.00	-40.49	-40.49	-45.42	-45.42	-43.73
Model composition							
Non-hydrogen atoms	21,876	21,804	22,308	22,176	21,624	21,552	21,312
Protein residues	2352	2352	2,328	2,328	2,316	2,316	2,304
Ligands	180	180	168	168	132	132	144
B factors (Å^2)							
Protein	40.46	37.12	51.09	50.04	41.92	47.13	59.04
Ligand	42.58	38.21	55.25	55.19	43.92	47.64	61.77
R.m.s. deviations							
Bond lengths (Å)	0.011	0.010	0.010	0.007	0.009	0.009	0.007
Bond angles (°)	0.824	0.835	0.825	0.821	0.934	1.073	0.816
Validation							
MolProbity score	0.80	1.03	1.03	1.03	1.13	1.22	0.98
Clashscore	0.91	2.49	2.45	2.46	2.90	3.93	2.07
Poor rotamers (%)	0.57	0.56	0.00	0.56	1.17	1.14	0.00
Ramachandran plot							
Favored (%)	97.92	98.44	98.11	98.95	98.41	98.94	98.40
Allowed (%)	2.08	1.56	1.89	1.05	1.59	1.06	1.60
Disallowed (%)	0.00	0.00	0.00	0.00	0.00	0.00	0.00

Supplementary Table 1. CryoEM Statistics. Summary of CryoEM data collection, refinement and model validation statistics. The ensemble CryoEM dataset was used to obtain the 1.90 Å resolution reconstruction and atomic models for Cx46 and Cx50, including 396 water molecules and 150 lipid acyl-chains. 3D classification was used to obtain the three PC classes, and associated atomic models for Cx46 and Cx50 (PC Class 1–3). Pre-processed and post-processed maps and associated masks from all datasets have been deposited to the EM databank (EMD-22358, EMD-22382, EMD-22390, EMD-22391). The original multi-frame micrographs have been deposited to EMPIAR (EMPIAR-10480). Coordinates for Cx50 and Cx46 atomic models have

been deposited to the Protein Data Bank (7JJP and 7JKC correspond to the high-resolution models, 7JLW and 7JMD correspond to the ~2.5 Å models from PC Class 1; 7JM9 and 7JN0 correspond to PC Class 2; and 7JMC and 7JN1 correspond to PC Class 3).

Supplementary Figure 1

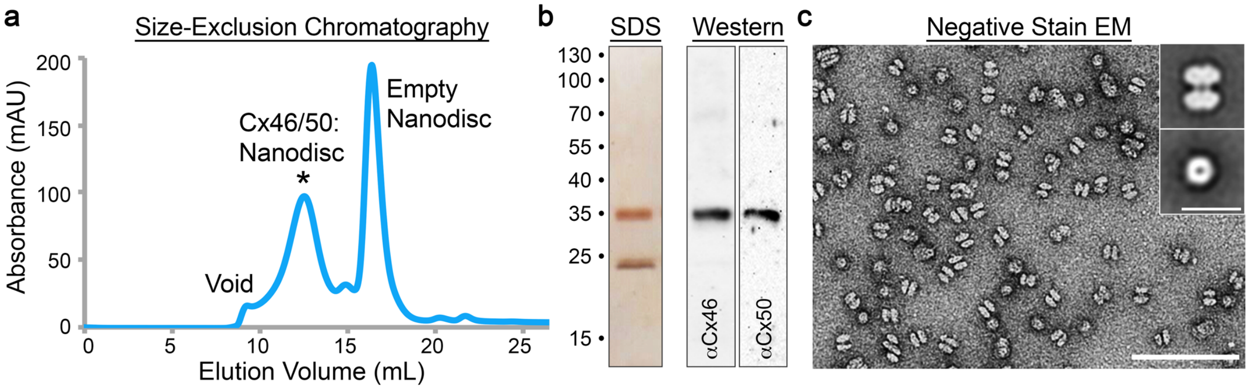

Supplementary Figure 1. Cx46/50 reconstitution into MSP1E1/DMPC lipid-nanodiscs and negative stain EM. a) Size-exclusion chromatography (SEC) trace monitored by UV absorbance at 280 nm. Peaks corresponding to Cx46/50 reconstituted into MSP1E1/DMPC nanodiscs (asterisk), empty nanodisc and void are indicated. **b)** SDS-PAGE (left) and western blot (right) of peak SEC fraction (labeled asterisk), with molecular weight markers indicated. MSP1E1 migrates as a ~24 kDa band (predicted MW ~27.5 kDa). Cx46 and Cx50 migrate together at ~38 kDa band, as expected from c-terminal truncation from core lens fiber cells[1], and confirmed by western blot (right). **c)** Electron micrograph of negatively stained particles from SEC fraction (labeled asterisk), with scale bar = 100 nm. *Inset*, shows representative 2D class averages of sideview (top) and top view (bottom), with scale bar = 20 nm. Particles display dumbbell-like structures corresponding to Cx46/50 gap junctions intercellular channels[1,2], embedded into a pair of ~10-11 nm wide nanodiscs (MSP1E1 nanodiscs have a predicted diameter of ~10.5 nm[3]).

Supplementary Figure 2

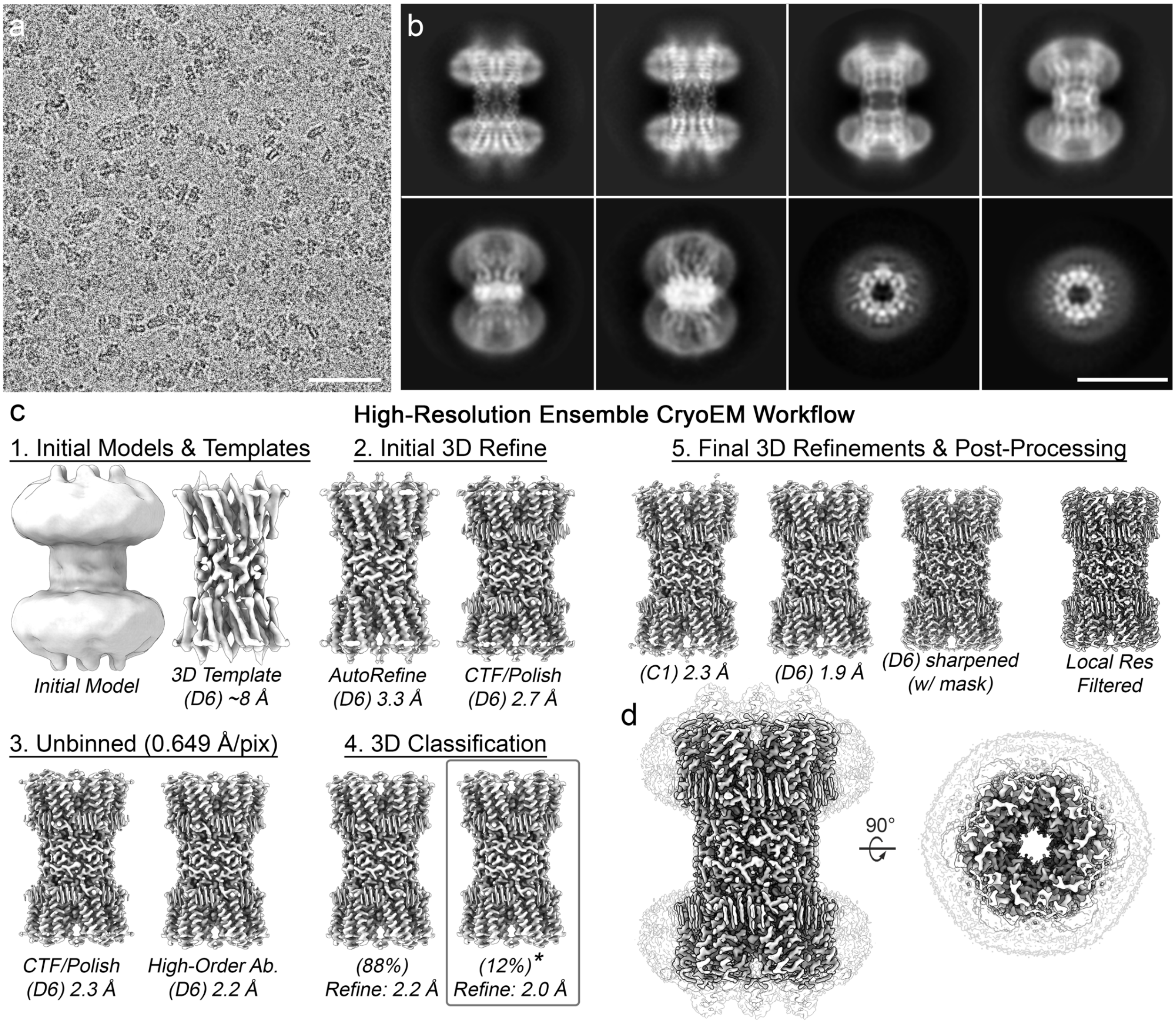

Supplementary Figure 2. CryoEM image processing workflow for 1.9 Å ensemble reconstruction of Cx46/50 in DMPC lipid nanodiscs. a) Representative CryoEM micrograph (dataset of 2088 movies) recorded on a Falcon III detector, with physical pixel size = 0.649 Å^2 and total dose of ~60 e$^-$ per Å^2. Scale bar = 50 nm. **b)** Representative 2D class averages. Scale bar = 10 nm**. c)** Image processing and 3D reconstruction workflow carried out in Relion[4,5], with representative maps at different stages of the image processing pipeline. Step 1) De-novo model generated in Relion (left) and initial 3D AutoRefinement with D6-symmetry (~8 Å resolution, 3.9 Å pixel size) (right), which was then filtered to 20 Å and used for 3D template auto-picking in Relion (resulting in ~1.2M particle picks, which were culled to ~228k “good” particles following multiple rounds of 2D classification and de-duplication). Step 2) Resulting 3D AutoRefine with D6-symmetry without masking (3.2 Å resolution, 0.97 Å pixel size) (left), and resulting map following

per particle CTF-refinement and polishing in Relion (2.7 Å) (right). Step 3) Particles were unbinned (pixel size 0.649 Å/pix, box size = 400 pix) and refined with per-particle CTF-correction and polishing (2.3 Å) (left), and further refinement of high-order aberration parameters in Relion v3.1-beta[4] (2.2 Å) (right). Step 4) Particles were de-duplicated, resulting in a set of ~221k particles, and subjected to 3D classification (two classes). Class 1 contained 88% of the particles and was further refined to 2.2 Å resolution (left). Class 2 contained 12% of the particles and was further refined to 2.0 Å resolution (right, asterisk). It is noted that the major differences between these two classes appears to be the distribution of particle defocus values, were the higher resolution class contains particles with lower defocus range (mean defocus = 1.20 ± 0.29 μm (s.d.)). Step 5) Particles belonging to Class 2 (~26k particles), were then subjected to multiple rounds of 3D Auto-refinement followed by per-particle CTF, aberration-correction and polishing, using successively larger box-sizes until no further improvement, resulting in a final reconstruction at 2.3 Å resolution (C1 symmetry) (left) and 1.9 Å resolution (D6 symmetry) (center, left). There were no clear conformational differences between the C1 and D6-symmetrized reconstructions. The D6-symmeterized map was then subjected to post-processing (b-factor sharpening) (center, right) and local-resolution filtering in Relion (right) for downstream analysis. **d)** Final reconstruction of Cx46/50 following local resolution filtering, used for atomic-modeling. Transparent silhouette displays the unmasked map at low-contour to illustrate the dimensions of the lipid nanodisc densities.

Supplementary Figure 3

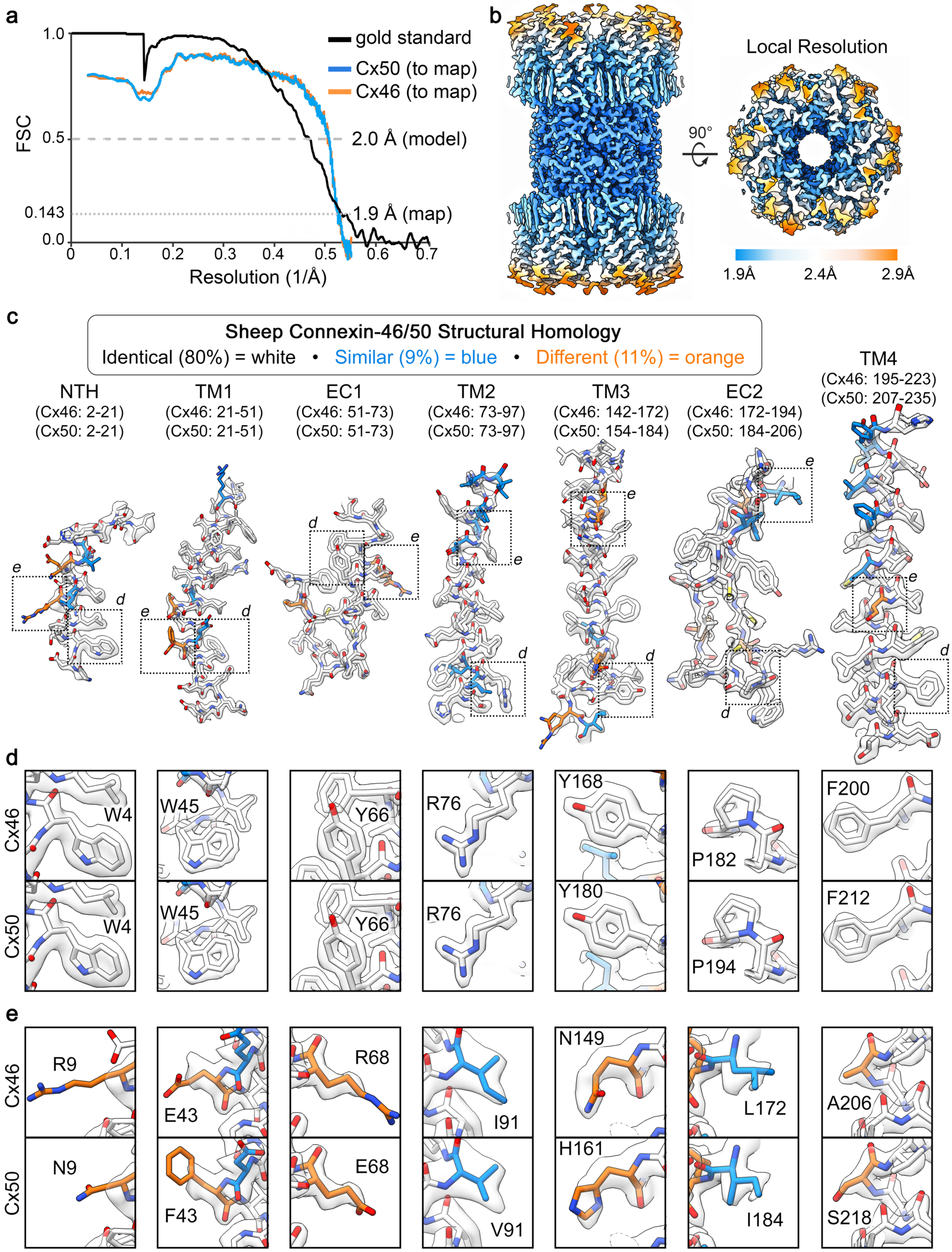

Supplementary Figure 3. Global and local resolution assessment of the 1.9 Å ensemble reconstruction of Cx46/50 in DMPC lipid nanodiscs. a) Fourier Shell Correlation (FSC) analysis obtained from the ensemble CryoEM map of Cx46/50 in DMPC lipid nanodiscs. Gold-standard FSC (black) of the final refined CryoEM map indicates a global resolution of 1.9 Å (0.143 cut-off). FSC curves comparing atomic models for Cx50 (blue) and Cx46 (orange) fit to the final CryoEM map display overall correlation at 2.0 Å (0.5 cut-off). **b)** Local resolution analysis of the final CryoEM map using Relion[4], displayed by colored surface (1.9 – 2.4 Å = blue – white; 2.4 – 2.9 Å = white – orange). **c)** Segmented CryoEM map with regions of the atomic models for sheep Connexin-46 (Cx46) and Connexin-50 (Cx50) fit to the local-resolution filtered map. Residue numbering for Cx46 and Cx50 is displayed above the corresponding segments for the n-terminal helix domain (NTH) domain, the transmembrane domains 1-4 (TM1-4) and extracellular domains 1-2 (EC1-2), Residues are colored according to the pair-wise sequence homology between sheep Cx46 and Cx50, as being identical (white, 80%), similar (blue, 9%) and different (orange, 11%), with all heteroatoms colored by standard scheme (oxygen – red; nitrogen – blue, sulfur – yellow). **d, e)** Windows show zoom-views corresponding to boxed regions of the segmented maps. **d)** Displays fits over representative regions where both Cx46/50 contain identical amino acids, where the high-resolution features are well-resolved. **e)** Displays fits over representative regions where the sequence of Cx46 and Cx50 differ, and where sidechain density is weaker and/or consistent with heterogeneity. This is presumably due to the heteromeric/heterotypic mixture of these isoforms[1,6,7] and the imposed averaging of two different sidechains in these areas, and/or to relative flexibility at these sites, as many of these same residues correspond to solvent/lipid exposed sidechains (e.g., R9/N9; E43/F43, R68/E68, I91/V91 and A206/S218).

Supplementary Figure 4

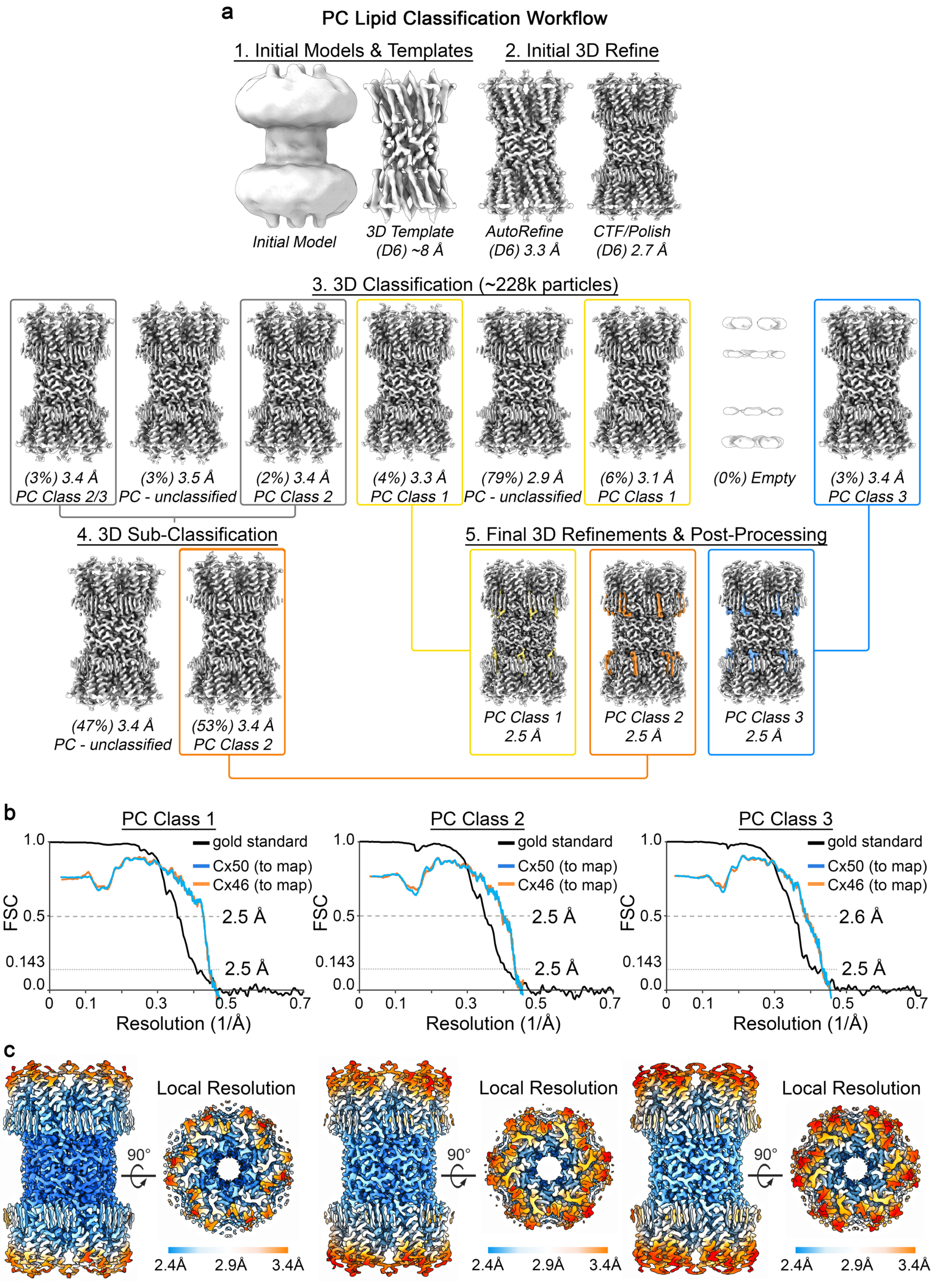

Supplementary Figure 4. Image processing and resolution assessment for 3D lipid-classification work-flow. a) Image process and 3D reconstruction workflow carried out in Relion for the analysis of PC lipid configuration/conformational heterogeneity, with representative maps at different stages of the image processing pipeline. Steps 1 and 2) are the same as described in Supplementary Fig. 2, which resulted in a 2.7 Å reconstruction from a dataset of ~228k "good" particles (right). Step 3) These particles were unbinned and re-extracted (0.649 Å/pix, 400 pixel box), and subjected to 3D classification (eight classes) without image alignment. Two of the eight classes yielded maps in which the lipid configuration was unambiguously resolved: assigned as PC Class 1 (yellow box), containing 9,190 particles (~4% of the data) and PC Class 3 (blue box), containing 6,944 particles (~3% of the data). Overlapping configurations were resolved in two of the other 3D classes (grey boxes). Step 4) The particles from these overlapping classes (grey boxes) were combined and subjected to a second round of 3D classification with two classes. This yielded one in which the lipid configuration was unambiguously resolved: assigned PC Class 2 (orange box), containing 6,075 particles (~3% of the data). Step 5) Particles assigned to PC Class 1 (left), PC Class 2 (center) and PC Class 3 (right) were separately subjected to a final round of 3D refinement and per-particle polishing, with D6 symmetry applied, resulting in final reconstructions ~2.5 Å resolution (Gold-Standard, 0.143 cut-off). **b)** Fourier Shell Correlation (FSC) analysis obtained for PC Class 1 (left), PC Class 2 (center) and PC Class 3 (right). Gold-standard FSC (black) of the final refined, masked and post-processed CryoEM map indicates a global resolution of 2.5 Å (0.143 cut-off). FSC curves comparing atomic models for Cx50 (blue) and Cx46 (orange) fit to the final CryoEM maps display overall correlation at 2.5–2.6 Å (0.5 cut-off). **c)** Local resolution analysis of the final CryoEM maps for PC Class 1 (left), PC Class 2 (center) and PC Class 3 (right) using Relion, displayed by colored surface (2.4 – 2.9 Å = blue – white; 2.9 – 3.4 Å = white – orange).

Supplementary Figure 5

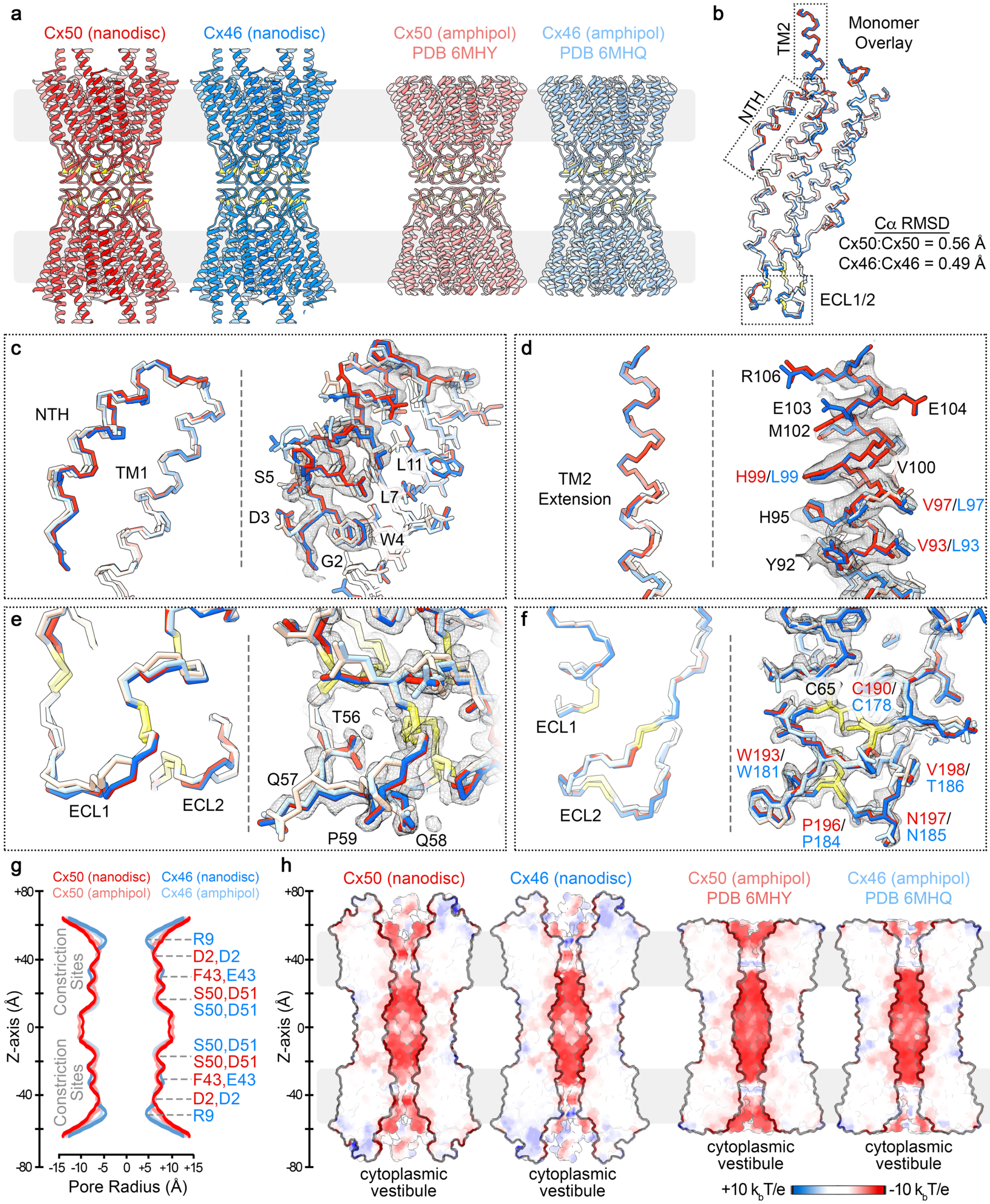

Supplementary Figure 5. Comparison of Cx46/50 structures determined in amphipol and lipid-nanodiscs. a) Ribbon structures of Cx50 (red) and Cx46 (blue) determined by CryoEM in lipid-nanodisc (left) and as previously determined in amphipol (right) with Cx50 (light red, PDB

6MHY) and Cx46 (light blue, PDB 6MHQ)[1]. Regions of lipid bilayer are indicated by light grey box. Conserved cysteine positions within the EC1/2 domains, involved in disulfide formation, are indicated in yellow. **b)** Cα traces over-laid for these four models, corresponding to a single subunit following super-positing (colored as in panel a). Cα r.m.s.d. following super-positioning is indicated for Cx50 (nanodisc) vs. Cx50 (amphipol) = 0.56 Å, and Cx46 (nanodisc) vs. Cx46 (amphipol) = 0.49 Å. **c–f)** Shows zoom views corresponding to the boxed regions in panel b. For each panel, (left) shows Cα trace and (right) shows all atom fit into the 1.9 Å CryoEM density map obtained from the nanodisc embedded structure, to show regions of improved fit to the experimental density map. Highlighted residues are indicated, and labels colored according to identity between the Cx50 and Cx46 isoforms (black – identical, red – Cx50 and blue – Cx46). **g)** Pore radius determined using HOLE[8], for experimental structures of Cx50-nanodisc (red), Cx46-nanodisc (blue), Cx50-amphipol (light red) and Cx46-amphipol (light blue). Locations corresponding to constriction sites are indicated, and residues contributing to these sites of constriction for both isoforms are labeled (Cx50 – red; Cx46 – blue). **h)** Cut-away surface representation of Cx50-nanodisc (left), Cx46-nanodisc (left center) and Cx50-amphipol (right center) and Cx46-amphipol (right), colored by coulombic potential (negative – red, neutral – white and positive – blue). This comparison illustrates the electrostatic environment of the permeation pathways and the extension of the intracellular vestibule that is resolved in the Cx46/50-nanodisc models, as compared to the previously described Cx46/50-amphipol models.

Supplementary Figure 6

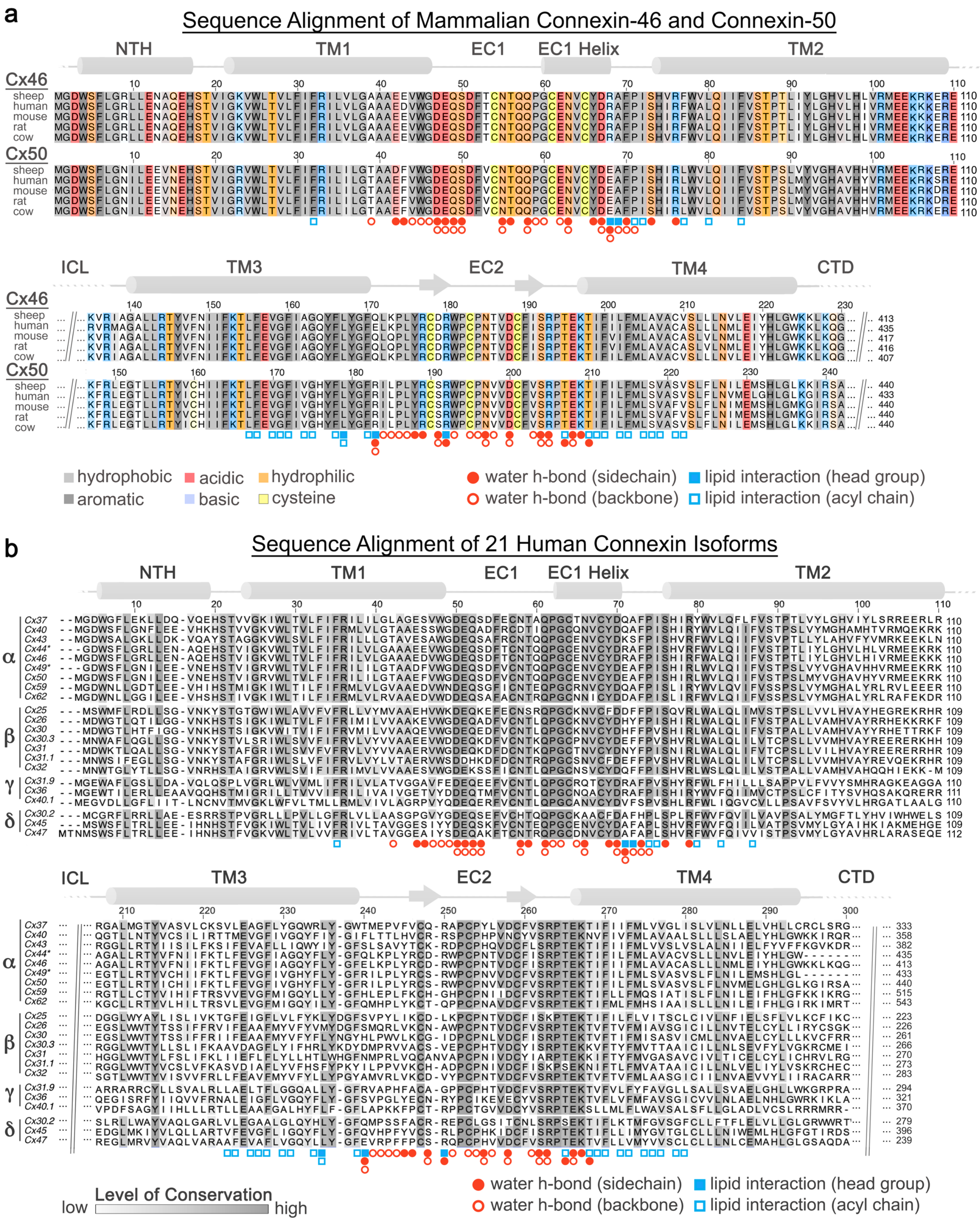

Supplementary Figure 6. Sequence alignment with annotated lipid and water binding sites.

a) Multiple sequence alignment of mammalian Cx46 and Cx50 isoforms with residues contributing

to lipid and/or water binding sites annotated (filled circle – water h-bonding with amino acid sidechain; open circle – water h-bonding with amino acid backbone) and (filled square – interaction involving lipid headgroup; open square – interaction involving lipid acyl chain). Primary sequence coloring corresponds to amino acid type (grey – hydrophobic; dark grey – aromatic; red – acidic; blue – basic; orange – hydrophilic; yellow – cysteine). Regions of sequence homology are indicated by the level of shading. Secondary structure and domain labels are indicated for the n-terminal helix (NTH), transmembrane helices (TM1-4) and extracellular domains (EC1-2). Regions lacking defined structure and of poor sequence homology within the intracellular loop (ICL) and c-terminal domain (CTD) have been omitted for clarity. Sheep and human Cx46 and Cx50 orthologs contain ~95% sequence identity (~98% similarity) over the structured regions of the protein. Numbering corresponds to the amino acid sequence of sheep Cx44 and Cx49 used in the main text. **b)** Multiple sequence alignment of 20 human connexin isoforms, with sheep Cx44 (Cx46 homolog) and Cx49 (Cx50 homolog) included for comparison. Isoforms are categorized by connexin family α, β, γ and δ. The orphan Cx23 was excluded from analysis. Regions of sequence homology are indicated by the level grey of shading. Annotations for lipid and water binding sites and secondary structural elements/domains are indicated as in panel a.

Supplementary Figure 7

a

	Connexin-50 (KCl)	Connexin-50 (NaCl)	Connexin-46 (KCl)	Connexin-46 (NaCl)
**Total Atoms**	356,838	356,838	357,545	357,545
Solvent	186,150	186,150	186,663	186,663
Lipids	131,806	131,806	131,806	131,806
Protein	38,484	38,484	38,712	38,712
Ions	398	398	364	364
Modelled Residues	2-109 ; 149-236	2-109 ; 149-236	2-109 ; 137-224	2-109 ; 137-224
Modifications to model	n-term acetylation	n-term acetylation	n-term acetylation	n-term acetylation
**Simulation Conditions**				
Simulation Box (Å)	147 x 147 x 174	147 x 147 x 174	147 x 147 x 174	147 x 147 x 174
Pressure (atm)	1	1	1	1
Temperature (K)	310	310	310	310
Time Step (fs)	2	2	2	2
Equilibration Time (ns)	30	30	30	30
Production Time (ns)	100	100	100	100
Production Replicas	2	2	2	2
Total Production Time (ns)	200	200	200	200

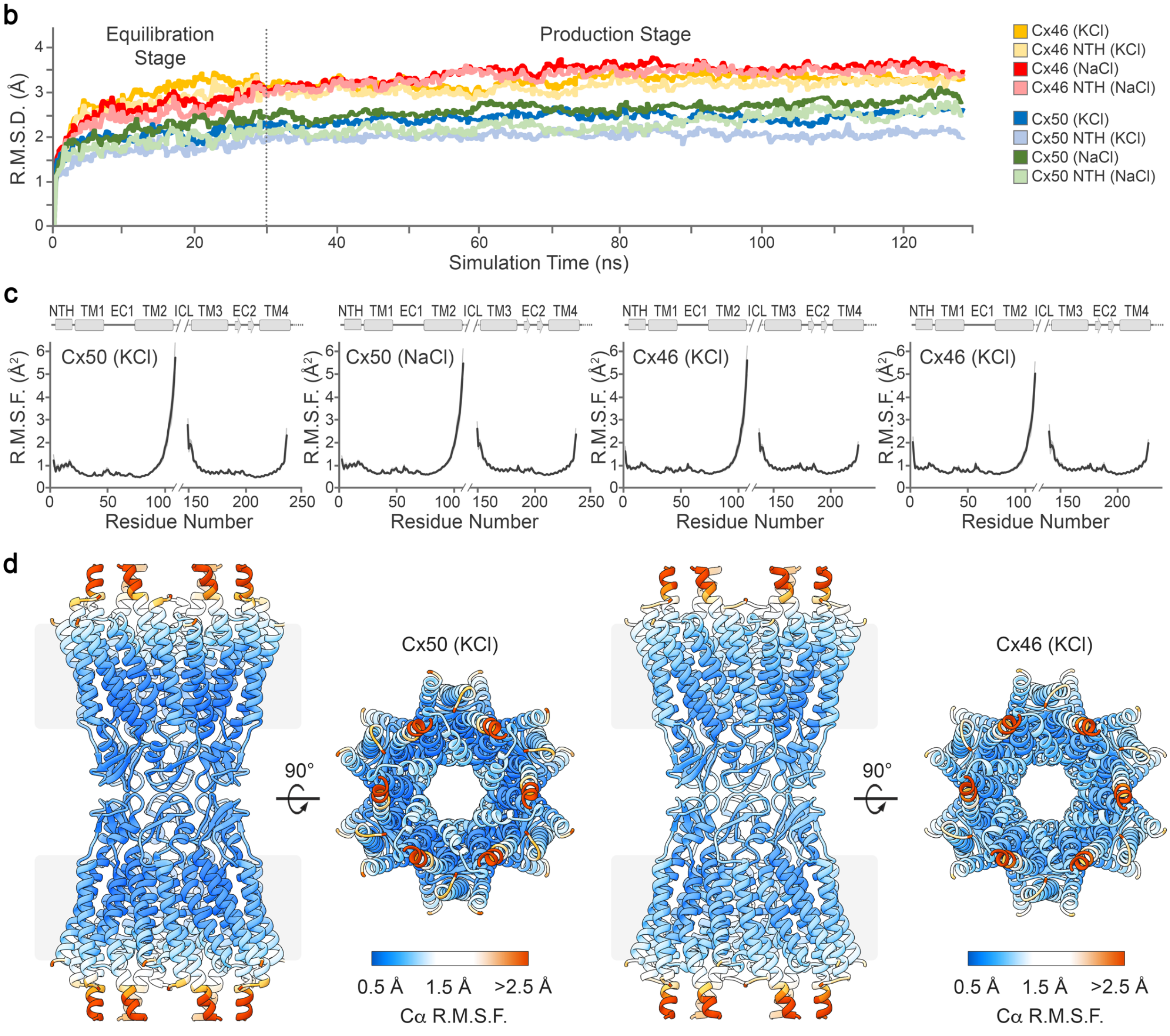

Supplementary Figure 7. Molecular dynamics setup and validation. a) Summary of molecular dynamics (MD) simulation setup and conditions. Each simulation was setup similarly, using and explicit solvent model containing either KCl (Cx50KCl; Cx46 KCl) or NaCl (Cx50 NaCl; Cx46 NaCl) in the cytoplasmic space, to match either cellular or in vitro conditions used for CryoEM studies, respectively. All simulations were conducted with NaCl in the extracellular space and using DMPC as the lipid system. Following minimization, all systems were equilibrated for 30 ns at 37° C, and multiple replicates (n = 2) of production (100 ns each) were acquired for analysis at 37° C. **b)** Cα root mean squared deviation (r.m.s.d.) analysis of equilibrium (0 – 30 ns) and production phases (30–130 ns) of the MD simulations, calculated with respect to the experimental starting structures, where Cx50 KCl (blue traces); Cx50 NaCl (green traces); Cx46 KCl (orange traces); Cx46 NaCl (red traces). Separate analysis for the n-terminal helix (NTH) domains are shown in lighter shades. **c)** Plot of average Cα root mean squared fluctuation (r.m.s.f.) during the production phase of the molecular dynamics (MD) simulations for Cx50 KCl (left), Cx50 NaCl (left center) Cx46 KCl (right center) and Cx46 NaCl (right). Averages are determined for the 12 subunits composing the intercellular channel, analyzed for both independent productions. Error bars (light grey shading) represent 95% confidence intervals (n = 24). Secondary structure and domain labels are indicated for the n-terminal helix (NTH), transmembrane helices (TM1-4), extracellular domains (EC1-2) and intracellular loop (ICL; not modeled). **d**) Representative r.m.s.f. values mapped to the experimental starting structures of Cx50 KCl (left) and Cx46 KCl (right). Colors correspond to r.m.s.f. amplitudes: < 0.5 Å (blue) – 1.5 Å (white) – 2.5 Å (red).

Supplementary Figure 8

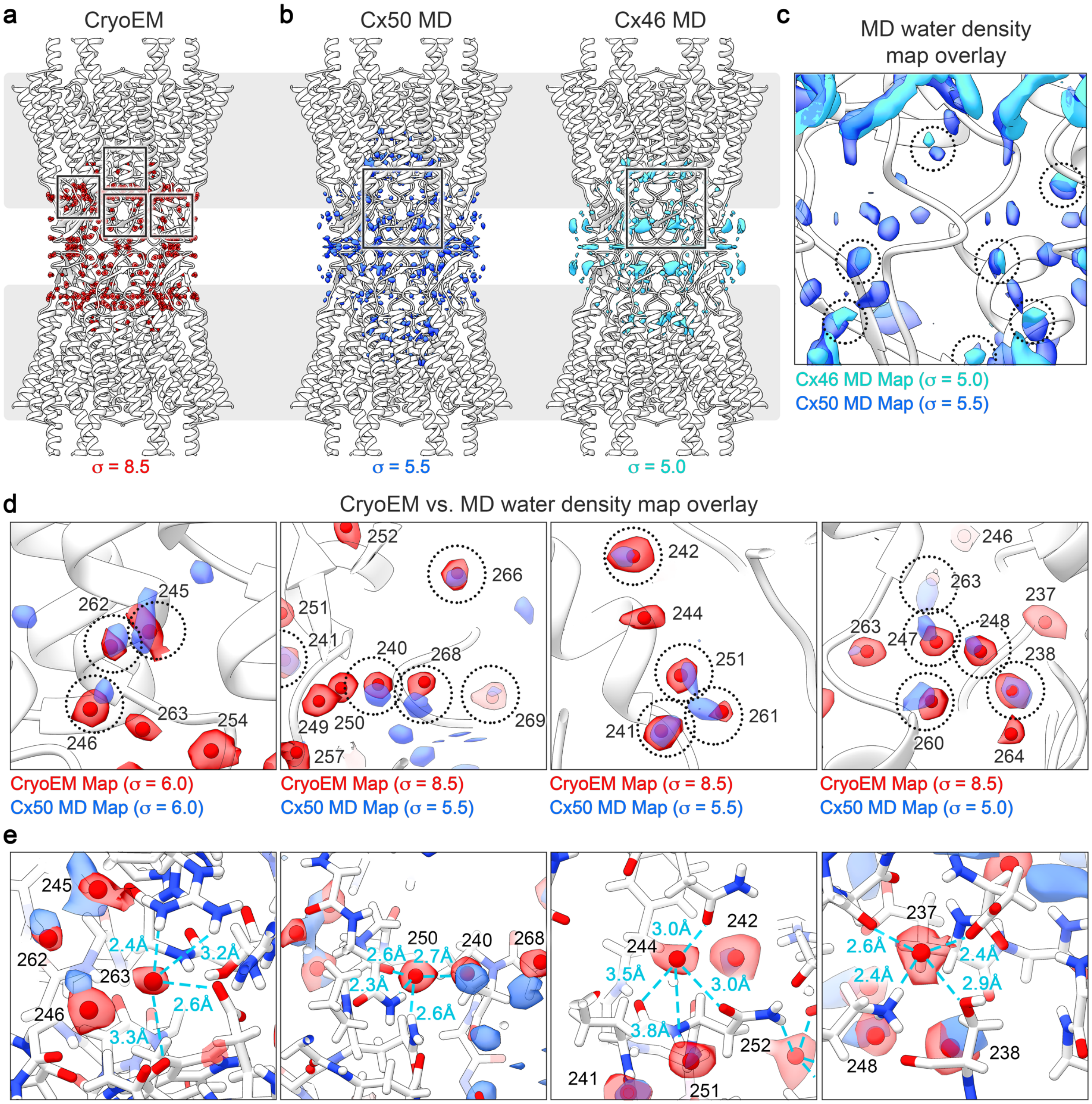

Supplementary Figure 8. Analysis of MD-based water density maps. a) Ribbon structure of Cx46/50 with segmented water densities from the ensemble CryoEM map (red density, threshold = 8.5 σ). **b)** Ribbon structures of Cx50/46 with overlaid time-averaged and symmetrized water density maps calculated from MD-simulation for Cx50 (left; blue density, threshold = 5.5 σ) and Cx46 (right; cyan density, threshold = 5.0 σ). **c)** Zoom view, corresponding to boxed regions in panels b and c, showing overlaid MD-based water densities. Representative regions of overlapping density are circled. **d)** Zoom views of boxed regions in panel a, showing representative regions of CryoEM water densities (red) overlaid the Cx50 MD-based water density

map (blue). Identities of modeled waters are indicated (using Cx50 numbering). Representative regions of overlapping density are circled. 76% of waters modeled into the CryoEM map show corresponding density in the MD-based water maps. Density map threshold values (σ) used for visualization in each panel are indicated. **e**) Alternative views to those presented in panel d, depicting some of the water densities observed by CryoEM that were not also resolved in the MD-based density maps. To validate these sites, the local coordination to sidechain and/or backbone hydrogen bond partners were assessed (donor/acceptor distances < 4 Å, blue dashed lines).

Supplementary Figure 9

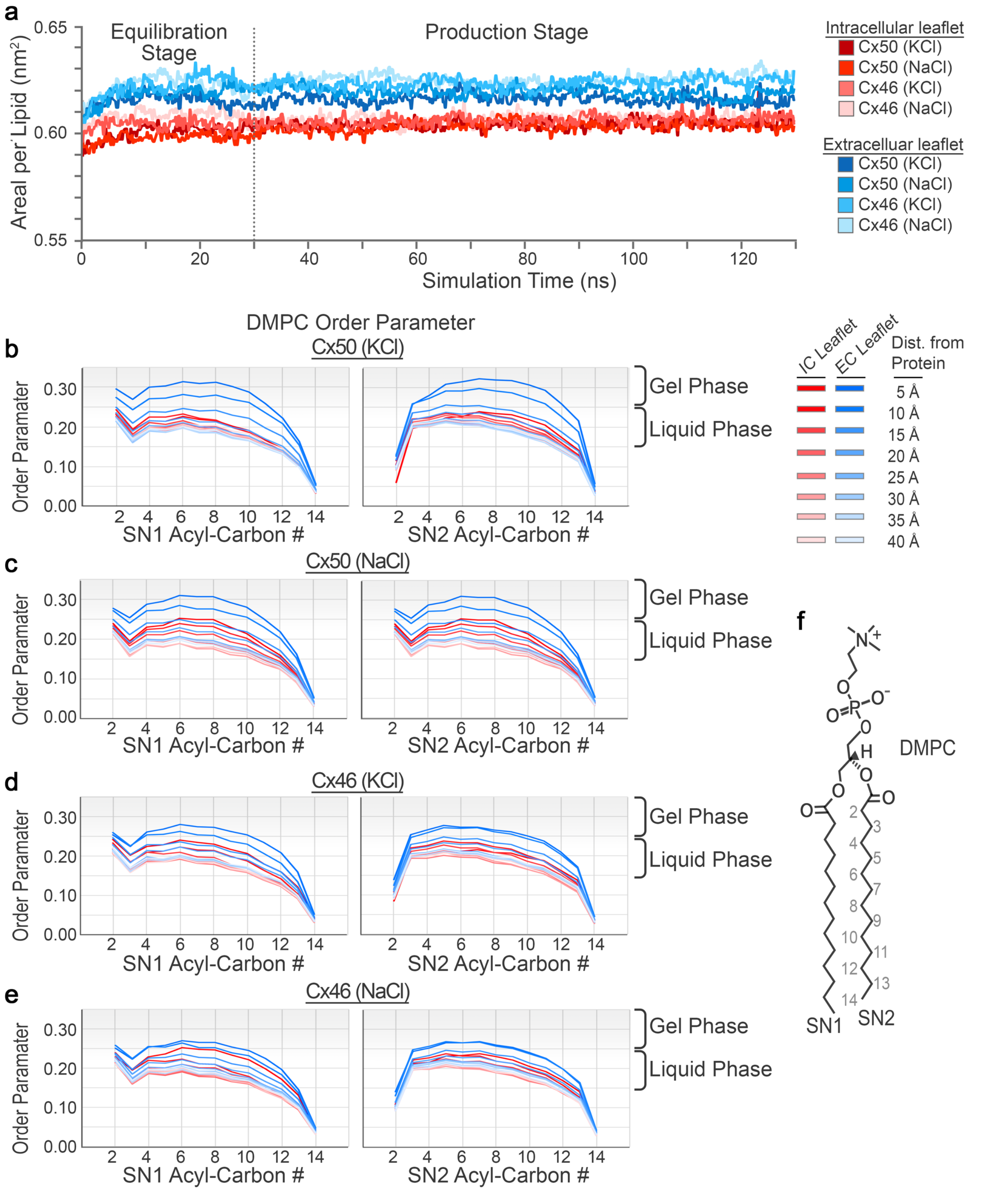

Supplementary Figure 9. Analysis of MD-based lipid dynamics. a) Lipid equilibration was monitored by analyzing the averaged area per lipid (nm^2) over the duration of MD-simulation. Traces correspond to lipids from the extracellular leaflets (blue shades) and intracellular leaflets

(red shades) for each system (Cx50 KCl, Cx50 NaCl, Cx46 KCl and Cx46 NaCl) are displayed. **b-e)** Averaged lipid order parameters calculated for the SN1 (left) and SN2 (right) acyl-chain C-H bonds (S_{CD}) for each system (panel b, Cx50 KCl; panel c, Cx50 NaCl; panel d, Cx46 KCl; and panel e, Cx46 NaCl). Traces correspond to lipids from the intracellular leaflets (red, IC leaflet) and extracellular leaflets (blue, EC leaflet), with dark to light shading showing the radial distance dependence from the surface of the protein (5 Å shells). **f)** Structure of dimyristoyl phosphatidylcholine (DMPC) with SN1 and SN2 acyl-chains labeled.

Supplementary References

1 Myers, J. B. *et al.* Structure of native lens connexin 46/50 intercellular channels by cryo-EM. *Nature* **564**, 372-377, doi:10.1038/s41586-018-0786-7 (2018).

2 Donaldson, P. & Kistler, J. Reconstitution of channels from preparations enriched in lens gap junction protein MP70. *J Membr Biol* **129**, 155-165 (1992).

3 Denisov, I. G., Grinkova, Y. V., Lazarides, A. A. & Sligar, S. G. Directed self-assembly of monodisperse phospholipid bilayer Nanodiscs with controlled size. *J Am Chem Soc* **126**, 3477-3487, doi:10.1021/ja0393574 (2004).

4 Zivanov, J., Nakane, T. & Scheres, S. H. W. Estimation of high-order aberrations and anisotropic magnification from cryo-EM data sets in RELION-3.1. *IUCrJ* **7**, 253-267, doi:10.1107/S2052252520000081 (2020).

5 Zivanov, J. *et al.* New tools for automated high-resolution cryo-EM structure determination in RELION-3. *eLife* **7**, doi:10.7554/eLife.42166 (2018).

6 Konig, N. & Zampighi, G. A. Purification of bovine lens cell-to-cell channels composed of connexin44 and connexin50. *J Cell Sci* **108 (Pt 9)**, 3091-3098 (1995).

7 Jiang, J. X. & Goodenough, D. A. Heteromeric connexons in lens gap junction channels. *Proc Natl Acad Sci U S A* **93**, 1287-1291 (1996).

8 Smart, O. S., Neduvelil, J. G., Wang, X., Wallace, B. A. & Sansom, M. S. HOLE: a program for the analysis of the pore dimensions of ion channel structural models. *J Mol Graph* **14**, 354-360, 376 (1996).
