# Supplementary material for: Connexin-46/50 in a dynamic lipid environment resolved by CryoEM at 1.9 Å
Source: Nat Commun. 2020 Aug 28;11:4331. doi: 10.1038/s41467-020-18120-5 (PMC7455559; doi:10.1038/s41467-020-18120-5)
Supplement: Supplementary file 8 — Reporting Summary [file 41467_2020_18120_MOESM8_ESM.pdf]

nature research

Corresponding author(s): Reichow, Steve L.

Last updated by author(s): 2020/08/04

Reporting Summary

Nature Research wishes to improve the reproducibility of the work that we publish. This form provides structure for consistency and transparency in reporting. For further information on Nature Research policies, see our Editorial Policies and the Editorial Policy Checklist.

Statistics

For all statistical analyses, confirm that the following items are present in the figure legend, table legend, main text, or Methods section.

n/a	Confirmed	
☐	☒	The exact sample size (*n*) for each experimental group/condition, given as a discrete number and unit of measurement
☐	☒	A statement on whether measurements were taken from distinct samples or whether the same sample was measured repeatedly
☐	☒	The statistical test(s) used AND whether they are one- or two-sided<br>*Only common tests should be described solely by name; describe more complex techniques in the Methods section.*
☒	☐	A description of all covariates tested
☒	☐	A description of any assumptions or corrections, such as tests of normality and adjustment for multiple comparisons
☐	☒	A full description of the statistical parameters including central tendency (e.g. means) or other basic estimates (e.g. regression coefficient) AND variation (e.g. standard deviation) or associated estimates of uncertainty (e.g. confidence intervals)
☒	☐	For null hypothesis testing, the test statistic (e.g. *F*, *t*, *r*) with confidence intervals, effect sizes, degrees of freedom and *P* value noted<br>*Give P values as exact values whenever suitable.*
☒	☐	For Bayesian analysis, information on the choice of priors and Markov chain Monte Carlo settings
☒	☐	For hierarchical and complex designs, identification of the appropriate level for tests and full reporting of outcomes
☒	☐	Estimates of effect sizes (e.g. Cohen's *d*, Pearson's *r*), indicating how they were calculated

Our web collection on statistics for biologists contains articles on many of the points above.

Software and code

Policy information about availability of computer code

Data collection	CryoEM: EPU (Thermo Fisher Scientific)
Data analysis	CryoEM Image Analysis Software: GCTF(v0.5), Relion(v3.0), Relion(v3.1-beta), EMAN2(v2.2); Atomic Modeling and Visualization: COOT(v0.8.6), Phenix(v1.17.1), Molprobity(v4.4), Chimera(v1.12), ChimeraX(v.0.91); Molecular Dynamics: VMD(v1.9.3), NAMD(v2.13), Solvate(v1.0.1)

For manuscripts utilizing custom algorithms or software that are central to the research but not yet described in published literature, software must be made available to editors and reviewers. We strongly encourage code deposition in a community repository (e.g. GitHub). See the Nature Research guidelines for submitting code & software for further information.

Data

Policy information about availability of data

All manuscripts must include a data availability statement. This statement should provide the following information, where applicable:

- Accession codes, unique identifiers, or web links for publicly available datasets
- A list of figures that have associated raw data
- A description of any restrictions on data availability

CryoEM density maps, including half-maps, pre- and post-processed maps and masks, have been deposited to the Electron Microscopy Data Bank (EMD-22358, EMD-22382, EMD-22390, EMD-22391). Coordinates for Cx50 and Cx46 atomic models have been deposited to the Protein Data Bank (PDB ID: 7JJP and 7JKC correspond to the high-resolution models, 7JLW and 7JMD correspond to the models from PC Class 1; 7JM9 and 7JN0 correspond to PC Class 2; and 7JMC and 7JN1 correspond to PC Class 3). The original multi-frame micrographs have been deposited to EMPIAR (EMPIAR-10480). MD trajectory files and MD-based density maps have been deposited to Zenodo (doi: 10.5281/zenodo.3951861).

Field-specific reporting

Please select the one below that is the best fit for your research. If you are not sure, read the appropriate sections before making your selection.

☒ Life sciences ☐ Behavioural & social sciences ☐ Ecological, evolutionary & environmental sciences

For a reference copy of the document with all sections, see nature.com/documents/nr-reporting-summary-flat.pdf

Life sciences study design

All studies must disclose on these points even when the disclosure is negative.

Sample size	CryoEM sample size were not predetermined. A dataset of 1,210,797 particles was collected from a total of 2087 micrographs. The size of this dataset was estimated based on instrument availability and expected target resolution.
Data exclusions	Single particle image data was excluded based on the absence of high-resolution features (e.g. alpha-helical transmembrane domains), which were conditions that had been pre-established based on previously published structural analysis.
Replication	CryoEM data processing was replicated by 3 independent experimentalists, and by using alternative CryoEM image processing software (CryoSPARC), yielding similar results. Molecular Dynamics trajectories were replicated twice for each system (100 ns simulations x 2 each).
Randomization	Single particle image data was split randomly into two groups and processed in the same way to calculate Fourier-shell correlation coefficients, in accordance to Gold Standard Methods. Samples were not further allocated into groups, outside of what is performed by the computational image analysis programs used in this work.
Blinding	Investigators were not blinded during data acquisition or analysis. Blinded studies in this case were not possible because the investigator performing the experiments and analysis also contributed to isolation of the specimen being analyzed.

Reporting for specific materials, systems and methods

We require information from authors about some types of materials, experimental systems and methods used in many studies. Here, indicate whether each material, system or method listed is relevant to your study. If you are not sure if a list item applies to your research, read the appropriate section before selecting a response.

Materials & experimental systems

n/a	Involved in the study
☐	☒ Antibodies
☒	☐ Eukaryotic cell lines
☒	☐ Palaeontology and archaeology
☒	☐ Animals and other organisms
☒	☐ Human research participants
☒	☐ Clinical data
☒	☐ Dual use research of concern

Methods

n/a	Involved in the study
☒	☐ ChIP-seq
☒	☐ Flow cytometry
☒	☐ MRI-based neuroimaging

Antibodies

Antibodies used	Antibodies were purchased from outside vendors. Primary antibodies directed against the n-terminal domain of Cx46 (rabbit, poly-clonal) where purchased from Acris (AP11570PU-N) and used for western blot analysis at a dilution of 1:500. Antibodies directed against the proximal n-terminal domain of Cx50 (rabbit, poly-clonal) where purchased from LSBio (LS-C116220).
Validation	The Cx46 antibody was validated by the manufacturer against the human isoform using ELISA and Immunohistochemistry. The Cx50 antibody was validated by the manufacturer by western blot analysis of mouse uterus lysate. Both antibodies were further validated by in-house western blot analysis using lens fiber cell lysates (sheep).
